# ZIKV prM hijacks PIM1 kinase for phosphorylation to prevent ubiquitin−mediated degradation and facilitate viral replication

**DOI:** 10.3389/fcimb.2024.1502770

**Published:** 2024-11-29

**Authors:** Yingying Ren, Yishuo Liu, Rui Pang, Gang Xu, Yining Lei, Hang Fai Kwok, Yingliang Wu, Zhijian Cao

**Affiliations:** ^1^ State Key Laboratory of Virology, College of Life Sciences, Wuhan University, Wuhan, Hubei, China; ^2^ Shenzhen Research Institute, Wuhan University, Shenzhen, Guangdong, China; ^3^ National “111” Center for Cellular Regulation and Molecular Pharmaceutics, Key Laboratory of Fermentation Engineering (Ministry of Education), Hubei University of Technology, Wuhan, Hubei, China; ^4^ Center for Evolution and Conservation Biology, Southern Marine Science and Engineering Guangdong Laboratory (Guangzhou), Guangzhou, Guangdong, China; ^5^ Department of Biomedical Sciences, Faculty of Health Sciences, University of Macau, Macao, Macao SAR, China

**Keywords:** ZIKV, prM, PIM1, phosphorylation, ubiquitination

## Abstract

**Introduction:**

Viral infection usually stimulates a variety of host cell factors to modulate the life cycle of the virus. PIM1, a serine/threonine protein kinase widely involved in cell proliferation, survival, differentiation and apoptosis, was recently reported to be upregulated by Zika virus (ZIKV) infection. However, how ZIKV-PIM1 interactions affect the viral life cycle are not fully understood.

**Methods and results:**

Here, we demonstrated that ZIKV replication was suppressed by the PIM1 kinase inhibitor SGI-1776 in both *wt* and *Ifnar1_-/-_* murine peritoneal macrophages, indicating that PIM1 functions independently of type I IFN signaling. Co-immunoprecipitation and GST pull-down assays revealed that the ZIKV structural protein precursor membrane (prM) interacted with PIM1. Moreover, we found that prM protein stability was enhanced by PIM1, which was attributed to its kinase activity. Mechanistically, we revealed that prM can undergo ubiquitin‒mediated proteolysis and the E3 ubiquitin ligase AMFR can target prM for degradation. Importantly, PIM1 catalyzed phosphorylation of prM at Ser101 and Thr107, and this phosphorylation prevented the proteasome-dependent degradation of prM by impairing its association with AMFR. Therefore, the S101/T107-D phosphorylation mimic mutant of prM was more resistant to PIM1-induced increases in cellular abundance.

**Discussion:**

These findings revealed PIM1 as a critical host factor that is advantageous to ZIKV and revealed that targeting the PIM1‒prM axis is a conducive strategy for controlling ZIKV infection.

## 1 Introduction

Zika virus (ZIKV) belongs to the *Orthoflavivirus* genus of the *Flaviviridae* family (Maslow and Roberts, 2020; Postler et al., 2023). It was first isolated from a sentinel monkey in Uganda in 1947, but only a few cases were reported until its emergence in South and Central America in 2015 (Giovannoni et al., 2020; Maslow and Roberts, 2020). In 2016, the World Health Organization declared that ZIKV caused a Public Health Emergency of International Concern (Hamel et al., 2015; Maslow and Roberts, 2020). Neural tissue, ocular tissue, placenta, uterus, and testis are susceptible to ZIKV according to previous human and animal studies (Miner and Diamond, 2017; Sirohi and Kuhn, 2017; Shaily and Upadhya, 2019). The symptoms of ZIKV infection are generally mild in adults, but it can cause fetal microcephaly when pregnant women are infected with ZIKV because ZIKV can traverse the placenta and induce robust infection in fetuses (Sirohi and Kuhn, 2017; Li et al., 2019; Inagaki et al., 2021).

ZIKV is an enveloped positive-sense, single-strand RNA virus (Giovannoni et al., 2020; Maslow and Roberts, 2020). The genomic RNA is approximately 10.8 kilobases long and encodes a polyprotein, which is processed to 3 structural proteins, including the capsid (C), the precursor membrane (prM) and the envelope (E), and 7 nonstructural proteins, including NS1, NS2A, NS2B, NS3, NS4A, NS4B and NS5 (Hamel et al., 2015; Sirohi and Kuhn, 2017; Goellner et al., 2023). The ZIKV structural protein prM participates in the formation of infectious virions (Sirohi and Kuhn, 2017; Goellner et al., 2023). During the maturation of infectious virions, prM is cleaved by the furin protease at the recognition site between the pr protein and the M protein on the trans-Golgi network (Stadler et al., 1997; Sirohi and Kuhn, 2017; Renner et al., 2021); thus, the inhibition of furin-mediated cleavage can suppress ZIKV replication (Imran et al., 2019). Even if prM is cleaved by furin protease, pr still binds to the E protein to obscure the fusion peptide on the E protein, preventing unproductive fusion until the mature virus is transported outside the cell, indicating the importance of prM in the assembly of infectious virions (Stadler et al., 1997; Yu et al., 2009; Renner et al., 2021). According to the cryo-EM structure of ZIKV, the stem and transmembrane regions of prM anchor the M protein to the lipid bilayer (Sirohi et al., 2016), and there are two functional cholesterol-binding motifs in the transmembrane region, which creates a platform to facilitate cholesterol-supported lipid exchange, supporting virus entry and promoting the assembly of infectious virions (Goellner et al., 2023). The prM protein of all ZIKV strains contains a single N-linked glycosylation site, which is necessary for the secretion of infectious Zika virions (Gwon et al., 2020). Moreover, the prM protein is tightly associated with the neurotoxicity of ZIKV infection, and a single mutation in prM can affect the pathogenicity of ZIKV (Yuan et al., 2017; Li et al., 2019; Inagaki et al., 2021). These studies indicate that the prM protein plays an important role in ZIKV replication. However, it is unclear whether the ZIKV prM protein interacts with host cell factors to affect its replication.

PIM1, a member of a highly conserved serine/threonine protein kinase family together with PIM2 and PIM3, was initially identified as a target for proviral activation by Moloney murine leukemia virus (Cuypers et al., 1984; Ma et al., 2007; Ko et al., 2022). PIM1 participates in cell proliferation, survival, differentiation, apoptosis, and tumorigenesis by phosphorylating various cellular substrates (Wang et al., 2001; Bachmann and Möröy, 2005; Nawijn et al., 2011; Yang et al., 2017). However, several lines of evidence have recently elucidated the role of PIM1 in virus replication and the virus-induced innate immune signaling pathway. PIM1 can inhibit virus-induced RIG-I- and MDA5-mediated IFN-β signaling to promote Sendai virus replication (Zhang et al., 2018). In contrast, reports suggest that PIM1 can promote IFN-β by interacting with interferon regulatory factor 3 (IRF3) to suppress virus replication (de Vries et al., 2015; Ko et al., 2022). Moreover, viruses can hijack PIM1 to promote replication. Enterovirus A71 (EV-A71) upregulates PIM1, which increases viral 2A protease-mediated eIF4G cleavage to increase viral internal ribosome entry site (IRES) activity and blocks the suppression of the IRES to promote enterovirus A71 replication (Zhou et al., 2019). Hepatitis C virus (HCV) utilizes PIM1 as a cell entry factor, and the HCV nonstructural 5A (NS5A) protein interacts with PIM1, thereby increasing PIM1 stability to promote HCV entry (Park et al., 2015). Notably, ZIKV infection upregulates PIM1 and exploits PIM1 to suppress type I IFN signaling activity (Zhou et al., 2021). Nevertheless, the interaction between PIM1 and ZIKV itself and the underlying molecular mechanism have not been extensively studied.

Here, we identified two ZIKV proteins, prM and NS1, that interact with PIM1 via co-immunoprecipitation assays and verified the direct interaction between prM and PIM1 via an *in vitro* GST pull-down assay. We also investigated the interaction regions within PIM1 and prM via co-immunoprecipitation assays in HEK293T cells using various truncation mutants of the PIM1 and prM proteins. Importantly, we found that impeding PIM1 kinase activity decreased prM protein stability and that PIM1 directly phosphorylated prM at amino acid residues Ser101 and Thr107. Further investigation revealed that this phosphorylation of prM catalyzed by PIM1 can impair the interaction of the E3 ubiquitin ligase AMFR with the prM protein, contributing to the inhibition of the ubiquitin−mediated proteolysis of the prM protein, thereby increasing the cellular abundance of the prM protein. We also found that the inhibition of PIM1 by SGI-1776 suppressed ZIKV replication in both *wt* and *Ifnar1^-/-^* primary murine peritoneal macrophages (mPMs), suggesting that the inhibition of PIM1 could suppress ZIKV replication in a type I IFN signaling-independent manner. Finally, our results showed that ZIKV can hijack the host cell factor PIM1 kinase to promote its own replication by phosphorylating its structural protein prM and therefore reducing prM degradation. Our findings highlight that targeting the PIM1−prM axis is a promising anti-ZIKV strategy.

## 2 Materials and methods

### 2.1 Mice

Wild-type C57BL/6 mice (male and female, six-week-old) were purchased from Wuhan Disease Control and Prevention Center. The type Ι interferon receptor-deficient A129 mice (*Ifnar1^-/-^*; on the C57BL/6 genetic background) were kindly provided by Professor Yu Chen from the College of Life Sciences at Wuhan University and bred in the animal facility under specific-pathogen-free conditions. All mouse experiments were approved by the Animal Experiment Center, College of Life Sciences, Wuhan University, and the Institutional Animal Care and Use Committee of Wuhan University (WDSKY0201707-2). All animal experiments were performed under the policies and recommendations of the institution and committee.

### 2.2 Cell culture and transfection

HEK293T cells and A549 cells were cultured in DMEM (Gibco) supplemented with 10% FBS (Gibco), 100 U/mL penicillin and 100 μg/mL streptomycin (Yeasen) at 37°C in 5% CO_2_. Murine peritoneal macrophages (mPMs) were isolated from six-week-old wild-type or *Ifnar1^-/-^* C57BL/6 mice and cultured in RPMI 1640 (Gibco) medium supplemented with 10% FBS (Gibco), 100 U/mL penicillin and 100 μg/mL streptomycin (Yeasen) at 37°C in 5% CO_2_. *Aedes albopictus* C6/36 cells were cultured in MEM (Gibco) supplemented with 10% FBS (Gibco), 100 U/mL penicillin and 100 μg/mL streptomycin (Yeasen) at 28°C in 5% CO_2_. For transfection, cells were transfected with the indicated constructs using ExFect Transfection Reagent (Vazyme) according to the manufacturer’s instructions.

### 2.3 Virus and amplification

The Zika virus Puerto Rico strain (PRVABC59) cDNA plasmid was kindly provided by Drs. Ren Sun and Danyang Gong at the University of California, Los Angeles, and the virus particles were prepared via a reverse genetic system. For amplification, C6/36 cells were infected with ZIKV at an MOI of 0.1, and the supernatants were harvested at 7 days post-infection, centrifuged at 8,000 rpm for 10 min at 4°C to remove cellular debris, and stored at −80°C as a stock. The virus titration was identified by detecting the TCID_50_ of ZIKV.

### 2.4 DNA constructs and cloning

The expression constructs for HA-PIM1 (WT, 65-313, 140-313, 1-177, 1-140), Flag-C, Flag-prM, Flag-pr, Flag-M, Flag-E, Flag-NS1, Flag-NS2B-3, Flag-NS4A, Flag-NS5, Myc-AMFR, Myc-RNF5, GST-PIM1, 6×His-PIM1, and 6×His-prM-CD (cytoplasmic domain of prM) were generated by amplifying the corresponding cDNA via PCR and cloning it into pKH3, pcDNA3.1 (+/−), pGEX-6p-1, pET-32a-c (+) or pET-28a (+) expression vectors. HA-PIM1-K67M and the mutants of Flag-prM (T96/S99/S101/T107-D, T96/S99/S101-D, T96/S99/T107-D, T96/S101/T107-D, S99/S101/T107-D, and S101/T107-D) were generated via site-directed mutagenesis.

### 2.5 Immunoblotting

The cells were washed with PBS two times and lysed with lysis buffer (1% SDS) supplemented with protease inhibitor cocktail (TargetMol; C0001). Proteins were separated by SDS−PAGE and transferred to nitrocellulose membranes (Whatman; 10401196). The membranes were blocked in 5% nonfat milk (BioFroxx; 1172GR500) for 2 h at room temperature and then incubated with the indicated primary antibodies overnight at 4°C. After being washed five times with TBST (20 mM Tris; 150 mM NaCl; 0.1% Tween-20; pH 7.6), the membranes were incubated with appropriate HRP-conjugated AffiniPure goat anti-mouse/rabbit IgG(H+L) secondary antibodies for 2 hours at room temperature. The immunoreactive bands were visualized via Clarity Western ECL Substrate (Bio-Rad; 1705061). Primary antibodies included DDDDK-tag Mouse mAb (MBL; M185-3L), DDDDK-Tag Rabbit mAb (ABclonal; AE063), Rabbit anti HA-Tag pAb (ABclonal; AE036), HA Tag Mouse Monoclonal antibody (Proteintech; 66006), Rabbit anti GST-Tag pAb (ABclonal, AE006), GST Tag Monoclonal antibody (Proteintech; 66001), Rabbit anti His-tag mAb (ABclonal; AE086), His-Tag Monoclonal antibody (Proteintech; 66005), Myc-Tag Rabbit mAb (ABclonal; AE070), MYC tag Monoclonal antibody (Proteintech; 60003), GAPDH Monoclonal antibody (Proteintech; 60004), Zika virus Envelope protein antibody (GeneTex; GTX133314), and Ubiquitin Rabbit mAb (ABclonal; A19686). The secondary antibodies used were HRP-conjugated AffiniPure goat anti-mouse or anti-rabbit IgG (Proteintech; SA00001). The intensity of the immunoblotting bands was determined via densitometry with ImageJ software.

### 2.6 Immunoprecipitation and ubiquitination assay

The cells were washed with PBS two times and lysed with IP buffer (150 mM NaCl; 59.50 mM HEPES; 1% Triton X-100; 10% glycerol; pH 7.2) supplemented with protease inhibitor cocktail (TargetMol; C0001) for 30 min on ice. After centrifugation for 15 min at 4°C, the supernatants were immunoprecipitated with Dynabeads Protein G (Invitrogen; 10003D) coated with the indicated primary antibodies overnight at 4°C. The bead-bound proteins were washed 5 times with IP buffer, and the immunoprecipitated proteins were boiled with 5× SDS−PAGE sample loading buffer (Biosharp; BL502A) for 10 min and analyzed by SDS−PAGE and immunoblotting. For analysis of the ubiquitination of prM, HEK293T cells were transfected with constructs expressing Flag-prM or other phosphorylation mimic mutants for 24 h and treated with 10 μM of the proteasome inhibitor MG132 (MedChemExpress; HY-13259) for 3 h before the cellular proteins were harvested. Then, the cell lysates were immunoprecipitated with an anti-Flag antibody and analyzed by immunoblotting with the indicated antibodies.

### 2.7 GST pull-down

The GST and GST-PIM1 recombinant proteins were expressed in *Escherichia coli* Rosetta(DE3) cells and purified via glutathione agarose resin (Solarbio; P2020). The 6×His-prM-CD recombinant protein was expressed in *Escherichia coli* Rosetta (DE3) cells and produced by denaturation with inclusion body solubilization buffer (20 mM Tris; 0.5 M NaCl; 20 mM imidazole; 10% glycerol; 8 M urea; pH 8.0), purification with Ni-IDA resin (GenScript; L00223I), and renaturation with renaturation buffer (50 mM Tris; 1 mM EDTA; 0.25 mM GSSG; 2.5 mM GSH; 200 mM L-arginine; 2.5 mM DTT; 0.1 M NaCl; 5% glycerol; pH 8.0). For GST pull-down, 15 μg of 6×His-prM-CD and 5 μg of GST or GST-PIM1 proteins were mixed and incubated with Dynabeads Protein G (Invitrogen; 10003D) coated with anti-GST antibodies overnight at 4°C in reaction buffer. The bead-bound proteins were washed 5 times with reaction buffer, and the complexes were boiled with 5× SDS−PAGE sample loading buffer (Biosharp; BL502A) for 10 min and analyzed by SDS−PAGE and immunoblotting.

### 2.8 *In vitro* kinase assay

The recombinant 6×His-PIM1 protein was expressed in *Escherichia coli* Rosetta(DE3) cells and purified via Ni-IDA resin (GenScript; L00223I). The recombinant proteins 6×His-PIM1 and 6×His-prM-CD were incubated in kinase buffer (Cell Signaling; 9802) for 1 h at 37°C, after which the reaction was terminated by boiling with 0.25 volumes of 5× SDS−PAGE sample loading buffer (Biosharp; BL502A) for 10 min. The proteins were separated by 12% SDS−PAGE, followed by Coomassie brilliant blue staining to identify the indicated proteins and ProQ Diamond phosphoprotein gel staining (Invitrogen; P33301) to detect the signal of phosphorylation according to the manufacturer’s instructions. In order to identify the exact phosphorylation sites of the prM protein, the specific band was cut and analyzed by tandem mass spectrometry (MS/MS) after Coomassie brilliant blue staining.

### 2.9 Cycloheximide chase assay

To examine the effects of PIM1 on the stability of the prM protein, HEK293T cells were transfected with the HA control, HA-PIM1 vector, or kinase-dead mutant, HA-PIM1-K67M vector, together with the Flag-prM vector in the absence or presence of 5 μΜ PIM kinase inhibitor SGI-1776 (MedChemExpress; HY-13287). At 24 h post-infection, the cells were treated with 50 μg/mL cycloheximide (MedChemExpress; HY-12320) for the indicated times before the cellular proteins were harvested. The proteins were analyzed by immunoblotting with the indicated antibodies.

### 2.10 Total RNA extraction and quantitative RT–PCR

The cells were washed with PBS two times, and total RNA was extracted via RNAiso Plus (Takara; 9109) according to the manufacturer’s instructions. The RNA quality and concentration were measured with a NanoDrop 2000 spectrophotometer (Thermo Fisher Scientific). The synthesis of cDNA was performed with a HiScript III 1st Strand cDNA Synthesis Kit (Vazyme; R312), and quantitative real-time PCR was performed via ChamQ SYBR qPCR Master Mix (Vazyme; Q331) on an Applied Biosystems 7500 HT Sequence Detection System according to the manufacturer’s instructions. Target mRNA expression levels were normalized to GAPDH mRNA expression levels. The sequences of primers used were as follows: GAPDH 5’-TGATGACATCAAGAAGGTGGTGAAG-3’ and 5’-TCCTTGGAGGCCATGTGGGCCAT-3’; ZIKV-E 5’-TTGTGGAAGGTATGTCAGGTG-3’ and 5’-ATCTTACCTCCGCCATGTTG-3’.

### 2.11 Isolation and culture of murine peritoneal macrophages

Six-week-old wild-type or *Ifnar1^-/-^* C57BL/6 mice were injected intraperitoneally with 1 mL of 4% thioglycollate medium (Solarbio; T105496) for 3 days to stimulate murine peritoneal macrophages. Five milliliters of cold PBS was infused into the peritoneal cavity to harvest peritoneal macrophages. The cells were washed twice with PBS, and erythrocyte were removed with red blood cell lysis buffer (Solarbio; R1010) according to the manufacturer’s instructions. Thereafter, the cells were resuspended in RPMI 1640 (Gibco) medium supplemented with 10% FBS (Gibco), 100 U/mL penicillin and 100 μg/mL streptomycin (Yeasen) and cultured in a 24-well flat-bottom plate at 37°C in 5% CO_2_.

### 2.12 Statistical analysis

All the data were obtained from at least three independent experiments. All the data are presented as the means ± SD. Data analysis was performed via the two-tailed unpaired Student’s *t* test or one-way ANOVA test with GraphPad Prism 8.0 software. *P* < 0.05 was considered significant.

## 3 Results

### 3.1 Screening of ZIKV proteins that interact with PIM1 and the promotion of PIM1 on prM cellular abundance

**Figure 1:**
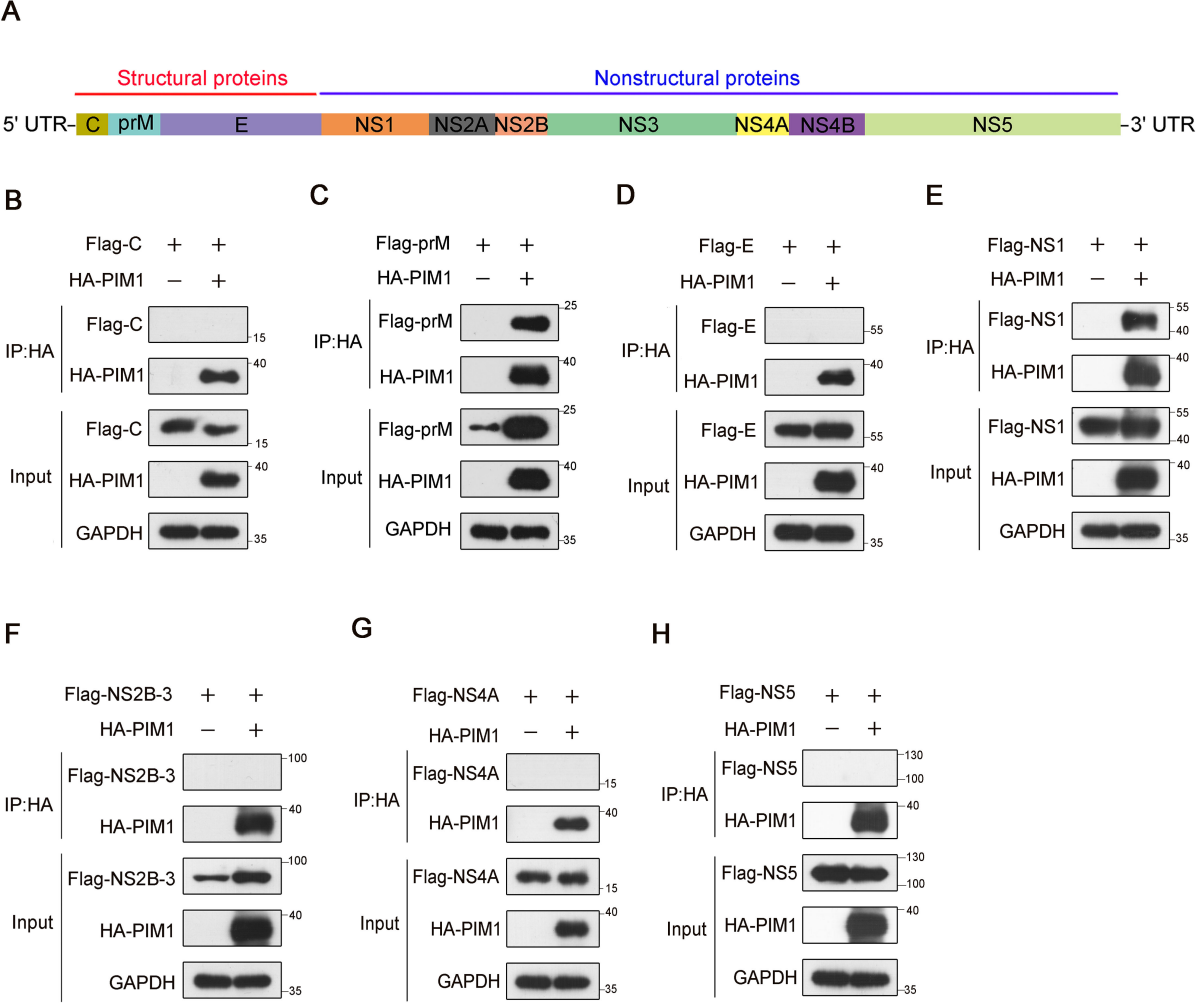
Screening of ZIKV proteins that interact with PIM1. **(A)** Schematic illustrates that ZIKV RNA encodes a polyprotein that is processed to 3 structural proteins (**(C)**, prM/M, and **(E)**) and 7 nonstructural proteins (NS1, NS2A, NS2B, NS3, NS4A, NS4B, and NS5). **(B–H)** HEK293T cells were transfected with constructs expressing HA-PIM1 and Flag-C **(B)**, Flag-prM **(C)**, Flag-E **(D)**, Flag-NS1 **(E)**, Flag-NS2B-3 **(F)**, Flag-NS4A **(G)**, or Flag-NS5 **(H)** for 24 h. The cell lysates were subjected to immunoprecipitation with an anti-HA antibody and analyzed by immunoblotting with the indicated antibodies.

The oncoprotein PIM1 kinase was revealed to inhibit the cellular type I IFN signaling pathway by vaguely regulating the phosphorylation of STAT1 or STAT2, which therefore promoted viral replication during ZIKV infection (Zhou et al., 2021). However, whether the promotion of PIM1 on ZIKV is dependent on its kinase activity and the phosphorylation of ZIKV proteins catalyzed by PIM1 is still unknown. To identify whether ZIKV proteins interact with PIM1, we performed co-immunoprecipitation in HEK293T cells transfected with constructs expressing HA-PIM1 and Flag-ZIKV proteins (C, prM, E, NS1, NS2B-3, NS4A, or NS5) (**Figure 1A**). As shown in **Figure 1**, two ZIKV proteins, prM and NS1, interact with PIM1 (**Figures 1C, E**). And ZIKV-C, ZIKV-E, ZIKV-NS2B-3, and ZIKV-NS5 proteins did not interact with PIM1 (**Figures 1B, D, F–H**). Interestingly, we also found that the overexpression of PIM1 sharply increased the cellular abundance of the prM protein (**Figure 1C**). To verify this enhanced phenomenon, HEK293T cells were co-transfected with the HA control or HA-PIM1 vector and the Flag-prM vector, and immunoblotting analysis of the cell extraction showed that the average promotion rate of prM cellular abundance induced by PIM1 was approximately 5.21 (**Supplementary Figures S1A, B**). Moreover, when different amounts of the HA-PIM1 plasmid were transfected into HEK293T cells, the expression of the prM protein in increased a dose-dependent manner (**Supplementary Figure S1C**). Besides, the overexpression of PIM1 could also significantly increase the protein level of prM under ZIKV infection (**Supplementary Figure S2**). We also performed another co-immunoprecipitation assay using an anti-Flag antibody to validate the interaction between PIM1 and prM (**Supplementary Figure S3**). These results indicate that the oncoprotein PIM1 kinase can specifically interact with the ZIKV prM protein and markedly increase prM cellular abundance.

### 3.2 Direct interaction of PIM1 and prM

**Figure 2:**
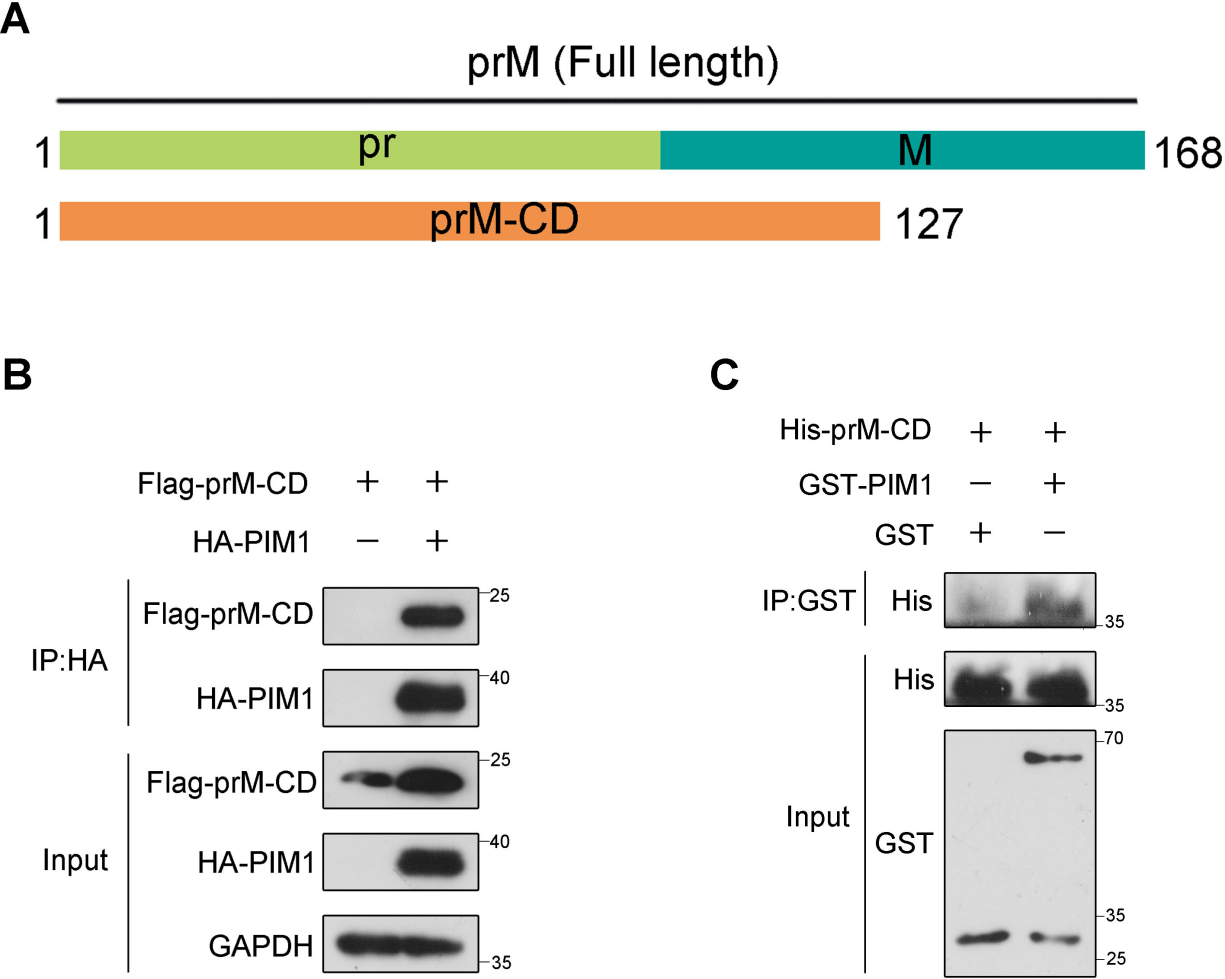
Direct interaction between PIM1 and prM. **(A)** Schematic illustration of constructs expressing the cytoplasmic domain of prM (prM-CD). **(B)** HEK293T cells were transfected with constructs expressing HA-PIM1 and Flag-prM-CD for 24 h. The cell lysates were subjected to immunoprecipitation with an anti-HA antibody and analyzed by immunoblotting with the indicated antibodies. **(C)** Bacterially purified recombinant 6×His-prM-CD was incubated in the absence or presence of bacterially purified recombinant GST-PIM1 with magnetic beads coated with anti-GST antibody. The beads were precipitated and subjected to immunoblotting with the indicated antibodies.

To determine whether the interaction between PIM1 and prM was direct, we constructed prokaryotic plasmids to express and purify recombinant human PIM1 or ZIKV prM in *Escherichia coli* cells. According to the cryo-EM structure of ZIKV (Sirohi et al., 2016), amino acids between 132 and 168 of prM anchor the protein into the lipid bilayer and ZIKV E protein, which cannot be recognized by cytoplasmic proteins in human cells. We also predicted the transmembrane domain of the prM protein with TMHMM software, which revealed that amino acids 1–127 aa of the prM protein could be recognized by human cytoplasmic proteins. Given that there is no serine or threonine in amino acids 128–132 aa of the prM protein, we constructed a plasmid for expressing and purifying amino acids 1–127 aa of the prM protein, named prM-CD (**Figure 2A**). We subsequently performed co-immunoprecipitation in HEK293T cells co-transfected with constructs expressing HA-PIM1 and Flag-prM-CD to test whether the interaction between PIM1 and prM still occurred when the C-terminus (amino acids 128–168 aa) of the prM protein was truncated. As shown in **Figure 2B**, prM-CD still interacted with PIM1, suggesting that the truncated protein prM-CD encompasses the interaction region responsible for PIM1 binding and thus could be used to perform GST pull-down to test the direct interaction between PIM1 and the prM protein. For the implementation of GST pull-down, we constructed prokaryotic expression vectors expressing recombinant GST-PIM1 and 6×His-prM-CD proteins respectively, and the vectors were subsequently transformed into *Escherichia coli* Rosetta(DE3) competent cells to produce the recombinant GST-PIM1 or 6×His-prM-CD proteins. The recombinant proteins GST-PIM1 purified by glutathione agarose resin and 6×His-prM-CD obtained by denaturation, purification with Ni-IDA resin, and renaturation were identified by SDS−PAGE (**Supplementary Figures S4A, B**). Thereafter, a GST pull-down assay was further carried out by incubating the recombinant 6×His-prM-CD with the full-length GST-PIM1. As shown in **Figure 2C**, 6×His-prM-CD specifically and directly interacted with GST-PIM1. Together, these data indicate that PIM1 directly interacts with the prM protein.

### 3.3 Identification of PIM1–prM interaction functional domains

**Figure 3:**
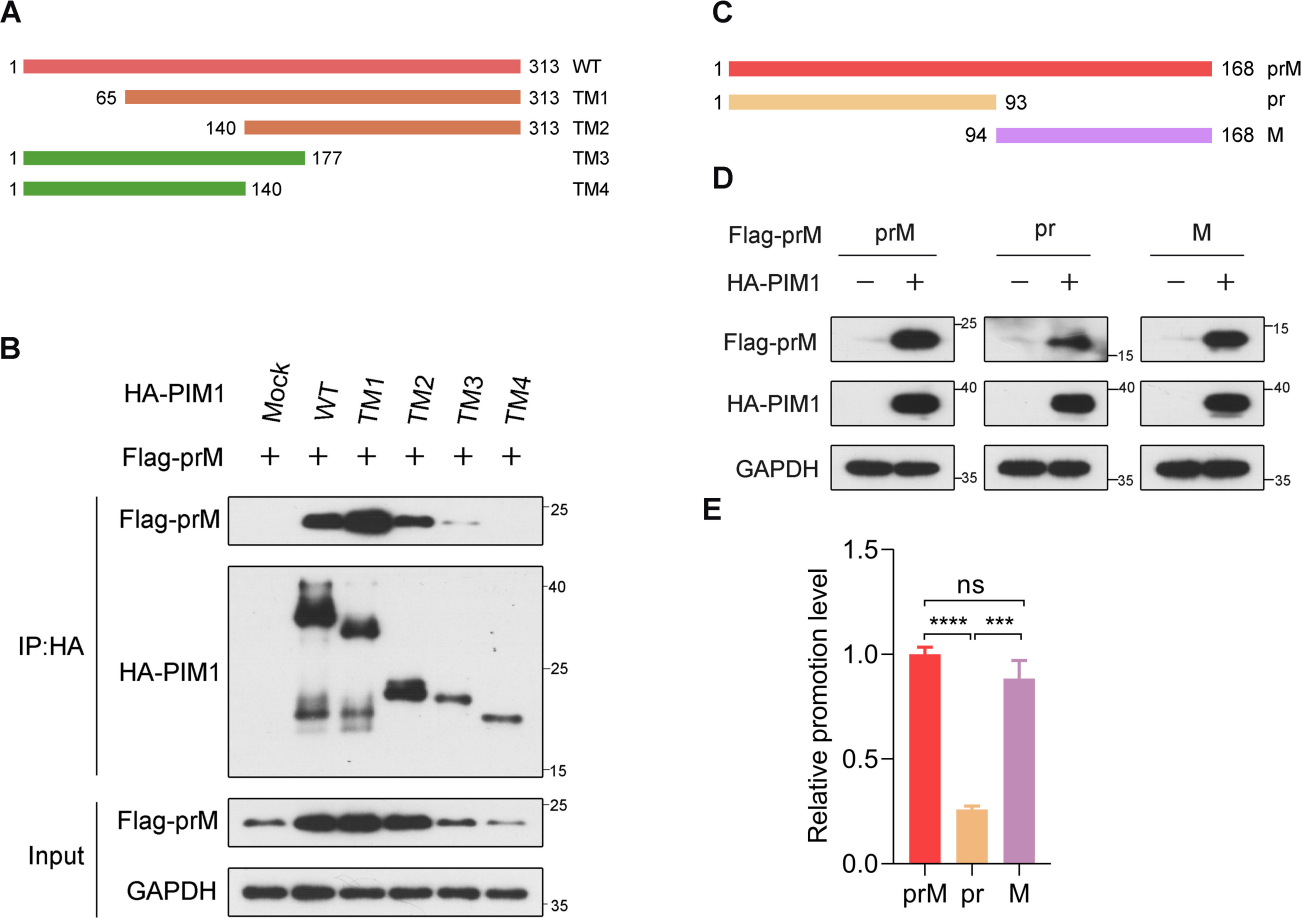
Identification of PIM1−prM interaction functional domains. **(A)** Schematic illustration of both the wild-type and mutant PIM1 expression constructs. **(B)** HEK293T cells were transfected with constructs expressing Flag-prM together with truncation mutants of HA-PIM1 (WT: 1-313 aa, TM1: 65-313 aa, TM2: 140-313 aa, TM3: 1-177 aa, and TM4: 1-140 aa) for 24 h. The cell lysates were subjected to immunoprecipitation with an anti-HA antibody and analyzed by immunoblotting with the indicated antibodies. **(C)** Schematic illustration of both the wild-type and mutant prM expression constructs. **(D)** HEK293T cells were transfected with the HA control or HA-PIM1 vector, together with the Flag-prM vector, Flag-pr vector, or Flag-M vector for 24 h. The cell lysates were analyzed by immunoblotting with the indicated antibodies. **(E)** Quantification of the relative promotion level normalized to prM in **(D)** (*n* = 3 independent experiments). The data are presented as the means ± SD. ***, *P* < 0.001; ****, *P* < 0.0001; ns, not significant. Statistical analysis was performed with a two-tailed unpaired Student’s *t* test.

To determine the region in PIM1 responsible for prM binding, we constructed various truncation mutants of HA-PIM1 (**Figure 3A**) according to previous studies (Ma et al., 2007; Park et al., 2015). HEK293T cells were transfected with each truncation mutant of the HA-PIM1 and Flag-prM vectors, and co-immunoprecipitation data demonstrated that prM interacted with full–length, 65–313 aa, 140–313 aa, and 1–177 aa of PIM1 but not with 1–140 aa of PIM1 (**Figure 3B**). On the basis of these results, we narrowed the region of PIM1 that interacted with prM to amino acid residues between 141 and 177 of PIM1, in which the amino acid sequences are involved in the construction of the ATP-binding pocket of PIM1 (Kumar et al., 2005; Qian et al., 2005).

Next, we investigated the prime region in the prM protein responsible for PIM1 functions. Between pr and M, a recognition site is cleaved by the furin protease in the trans-Golgi network (Renner et al., 2021). For this reason, we constructed plasmids expressing the Flag-pr and Flag-M proteins (**Figure 3C**). To detect the primary functional region of prM, HEK293T cells were transfected with constructs expressing HA control or HA-PIM1 and Flag-prM, Flag-pr or Flag-M. Immunoblotting analysis revealed that the promotion effects of PIM1 on the cellular abundance of prM and M proteins were barely discriminative, whereas the promotion effect of PIM1 on the cellular abundance of pr domain was markedly reduced relative to those of prM and M (**Figures 3D, E**). These data suggest that PIM1 mainly functions on M domain to increase the prM cellular abundance.

### 3.4 The dependence of the kinase activity of PIM1 to the prM cellular abundance promotion

**Figure 4:**
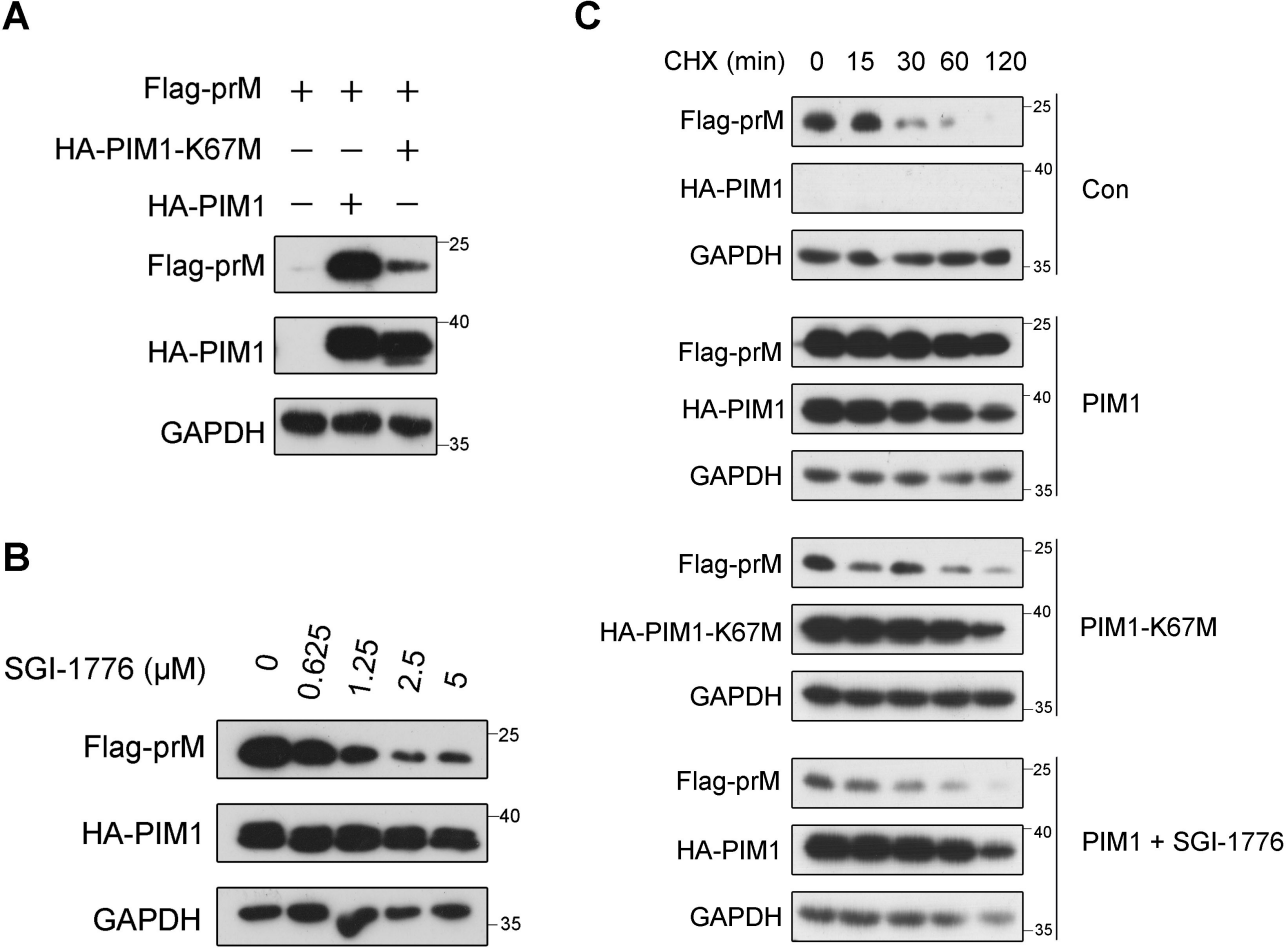
Kinase activity dependence of PIM1 promotion on prM cellular abundance. **(A)** HEK293T cells were transfected with the HA control, HA-PIM1 vector, or the kinase-dead mutant, HA-PIM1-K67M vector, together with the Flag-prM vector for 24 h. The cell lysates were analyzed by immunoblotting with the indicated antibodies. **(B)** HEK293T cells were transfected with constructs expressing HA-PIM1 and Flag-prM, and treated with the indicated concentration of SGI-1776 for 24 h. The cell lysates were analyzed by immunoblotting with the indicated antibodies. **(C)** HEK293T cells were transfected with the HA control, HA-PIM1 vector, or the kinase-dead mutant, HA-PIM1-K67M vector, together with the Flag-prM vector in the absence or presence of 5 μΜ SGI-1776. At 24 h after transfection, the cells were treated with 50 μg/mL CHX for the indicated times before the cellular proteins were harvested. The proteins were analyzed by immunoblotting with the indicated antibodies.

As shown in **Supplementary Figure S1C**, the level of prM sharply increased when the expression of PIM1 increased. To determine whether the up-regulation of prM by PIM1 is dependent on PIM1 kinase activity, HEK293T cells were transfected with wild-type PIM1 or the kinase-dead mutant PIM1-K67M (Yang et al., 2017). As a result, immunoblotting analysis revealed that PIM1-K67M counteracted the PIM1-mediated up-regulation of prM (**Figure 4A**). Since SGI-1776 binds the ATP-binding pocket of the PIM family, it specifically inhibits PIM kinase activity (Blanco-Aparicio and Carnero, 2013). As shown in **Figure 4B**, the prM cellular abundance induced by PIM1 gradually decreased as the concentration of SGI-1776 increased, whereas SGI-1776 did not influence the level of prM without PIM1 application (**Supplementary Figure S5A**). Moreover, we also observed that the level of PIM1 gradually decreased as the concentration of SGI-1776 increased (**Figure 4B**), which was further confirmed by SGI-1776 treatment of PIM1 individually (**Supplementary Figure S5B**). Together, we presume that SGI-1776 has a dual-effect on prM levels affected by PIM1. One is to weaken the kinase activity of PIM1. The other is to decrease the cellular abundance of PIM1, which was supported by the previously reported conclusion that PIM1 stability decreases when SGI-1776 binds the ATP-binding pocket of PIM1 (Polier et al., 2013; Taniguchi et al., 2014). These results preliminarily show that the kinase activity of PIM1 is necessary for the up-regulation of prM induced by PIM1.

To further evaluate the necessity of the kinase activity of PIM1 for the up-regulation of prM, a cycloheximide (CHX) chase assay was performed in HEK293T cells transfected with the HA control, HA-PIM1 vector, or kinase-dead mutant HA-PIM1-K67M vector together with the Flag-prM vector in the absence or presence of SGI-1776. Since CHX is an eukaryotic protein synthesis inhibitor, the half-life of proteins can be evaluated with CHX treatment. As shown in **Figure 4C**, the half-life of prM increased with the expression of wild-type PIM1, suggesting that the effect of PIM1 on the increase in prM stability occurs in the post-translational stage. In contrast, there was no obvious effect of PIM1-K67M or SGI-1776 on the half-life of prM, further indicating that the kinase activity of PIM1 is necessary for the up-regulation of prM induced by PIM1.

### 3.5 Direct phosphorylation of S101 and T107 of prM by PIM1

**Figure 5:**
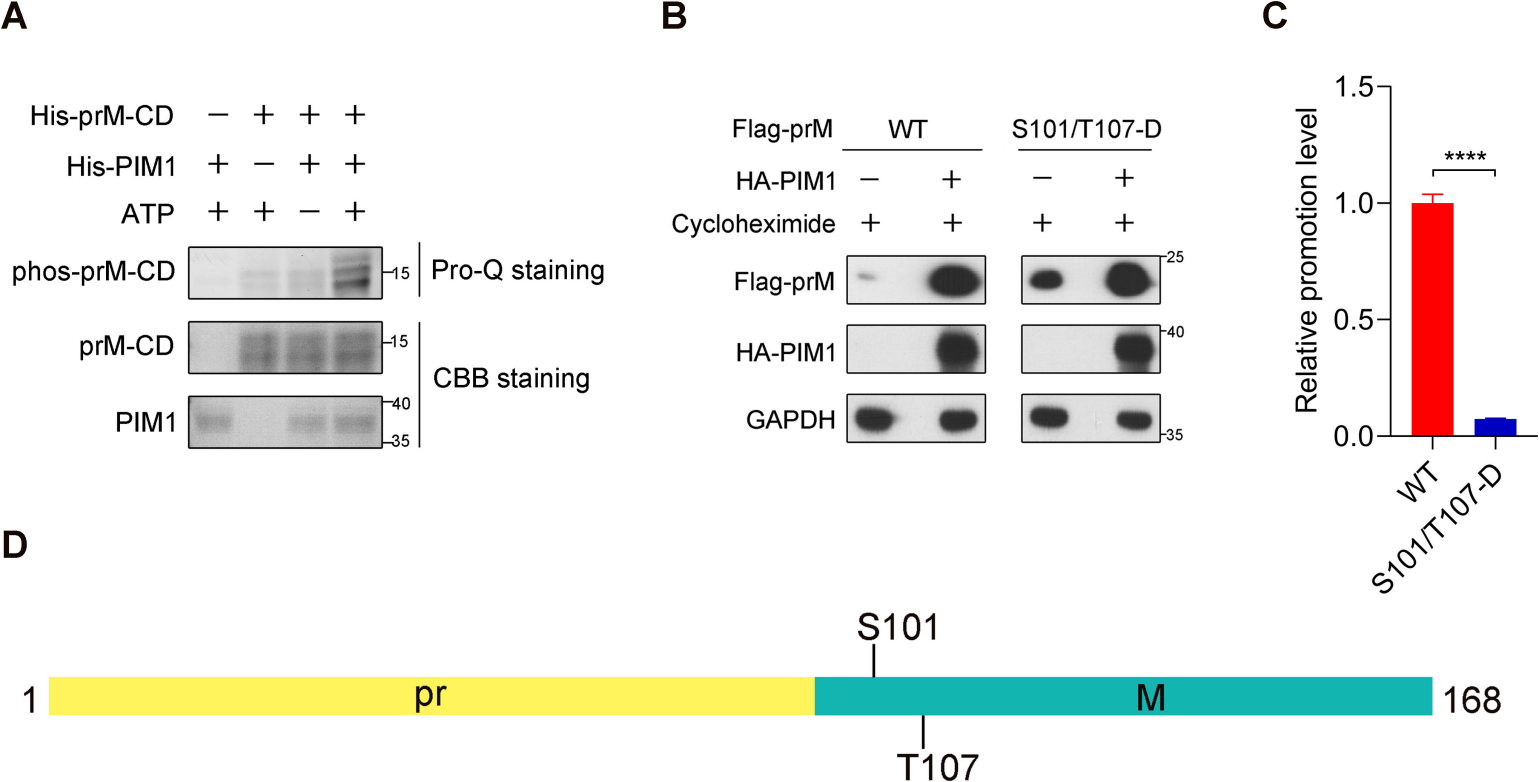
Direct phosphorylation of prM by PIM1. **(A)** An *in vitro* kinase assay using recombinant PIM1 and prM-CD was performed. The indicated proteins were detected by Coomassie brilliant blue staining, and the signal of phosphorylation was detected by ProQ Diamond phosphoprotein gel staining. **(B)** HEK293T cells were transfected with the HA control or HA-PIM1 vector, together with the Flag-prM vector or its phosphorylation mimic mutants. At 24 h post-infection, the cells were treated with 50 μg/mL CHX for 1 h before the cellular proteins were harvested. The proteins were analyzed by immunoblotting with the indicated antibodies. **(C)** Quantification of the relative promotion level in **(B)** (n = 3 independent experiments). The data are presented as the means ± SD. ****, *P* < 0.0001. Statistical analysis was performed with a two-tailed unpaired Student’s *t* test. **(D)** Schematic illustration of the exact phosphorylation sites of prM catalyzed by PIM1.

Because of the direct interaction between PIM1 kinase and the prM protein (**Figure 2C**) and the necessity of the kinase activity of PIM1 for prM up-regulation (**Figure 4**), we hypothesized that PIM1 may directly phosphorylate prM to regulate its stability. To detect whether prM was directly phosphorylated by PIM1, we constructed pET-28a-PIM1 and pET-28a-prM-CD prokaryotic expression vectors to produce recombinant 6×His-PIM1 and 6×His-prM-CD proteins with short tags, which were identified by SDS−PAGE, as shown in **Supplementary Figure S6**, to perform an *in vitro* kinase assay. ProQ phosphoprotein gel staining confirmed that prM was directly phosphorylated by PIM1 (**Figure 5A**). To further identify the specific sites of prM phosphorylated by PIM1, 6×His-prM-CD was recovered from the gel stained with Coomassie brilliant blue and analyzed by mass spectrometry. As shown in **Supplementary Figure S7A**, mass spectrometry analysis revealed that S8, Y13, T96, S99, S101, T107 and T119 of prM were phosphorylated by PIM1 *in vitro*. Because of the barely discriminative promotion effects of PIM1 on the cellular abundance of prM and M proteins and the markedly reduced promotion effect of PIM1 on the cellular abundance of pr domain (**Figures 3D, E**), we supposed that pr is not the predominant domain recognized by PIM1 and thus excluded the possible phosphorylation sites S8 and Y13 on the pr domain. According to the cryo-EM structure of ZIKV, the stem (114–132 aa) and transmembrane (133–168 aa) regions of prM anchor the M protein to the lipid bilayer, whereas the region encompassing amino acids 94 to 113 of prM can be recognized by cytoplasmic proteins in the process of ZIKV replication (Sirohi et al., 2016). For these reasons, we narrowed the phosphorylation sites of prM to T96, S99, S101 and T107, as displayed in **Supplementary Figure S7B**. To identify the exact phosphorylation sites of the prM protein, we constructed a series of mutants of Flag-prM (T96/S99/S101/T107-D; T96/S99/S101-D; T96/S99/T107-D; T96/S101/T107-D; S99/S101/T107-D) to detect the promotion efficiency of PIM1 on prM cellular abundance. Thereafter, HEK293T cells transfected with the HA control or HA-PIM1 vector and the Flag-prM vector or its phosphorylation mimic mutants were treated with the eukaryotic protein synthesis inhibitor CHX. Immunoblotting analysis revealed that the promotion efficiency induced by PIM1 was significantly restored compared with that of the T96/S99/S101/T107-D mutant when S101 or T107 could be recognized by PIM1, whereas there was no obvious change in the promotion efficiency when S101 and T107 could not be recognized by PIM1, indicating that S101 and T107 were the major phosphorylation sites of the prM protein (**Supplementary Figures S7C, D**). To verify these two phosphorylation sites, we constructed an S101/T107-D mutant of Flag-prM and transfected it into HEK293T cells together with the HA control or HA-PIM1 vector. Immunoblotting analysis revealed that the promotion efficiency of the S101/T107-D mutant induced by PIM1 was significantly lower than that of the wild-type prM, which was consistent with the above results (**Figures 5B, C**). These data show that PIM1 directly phosphorylates prM at S101 and T107 to increase prM cellular abundance (**Figure 5D**).

### 3.6 Inhibitory effect of prM phosphorylation on its ubiquitin−mediated degradation

**Figure 6:**
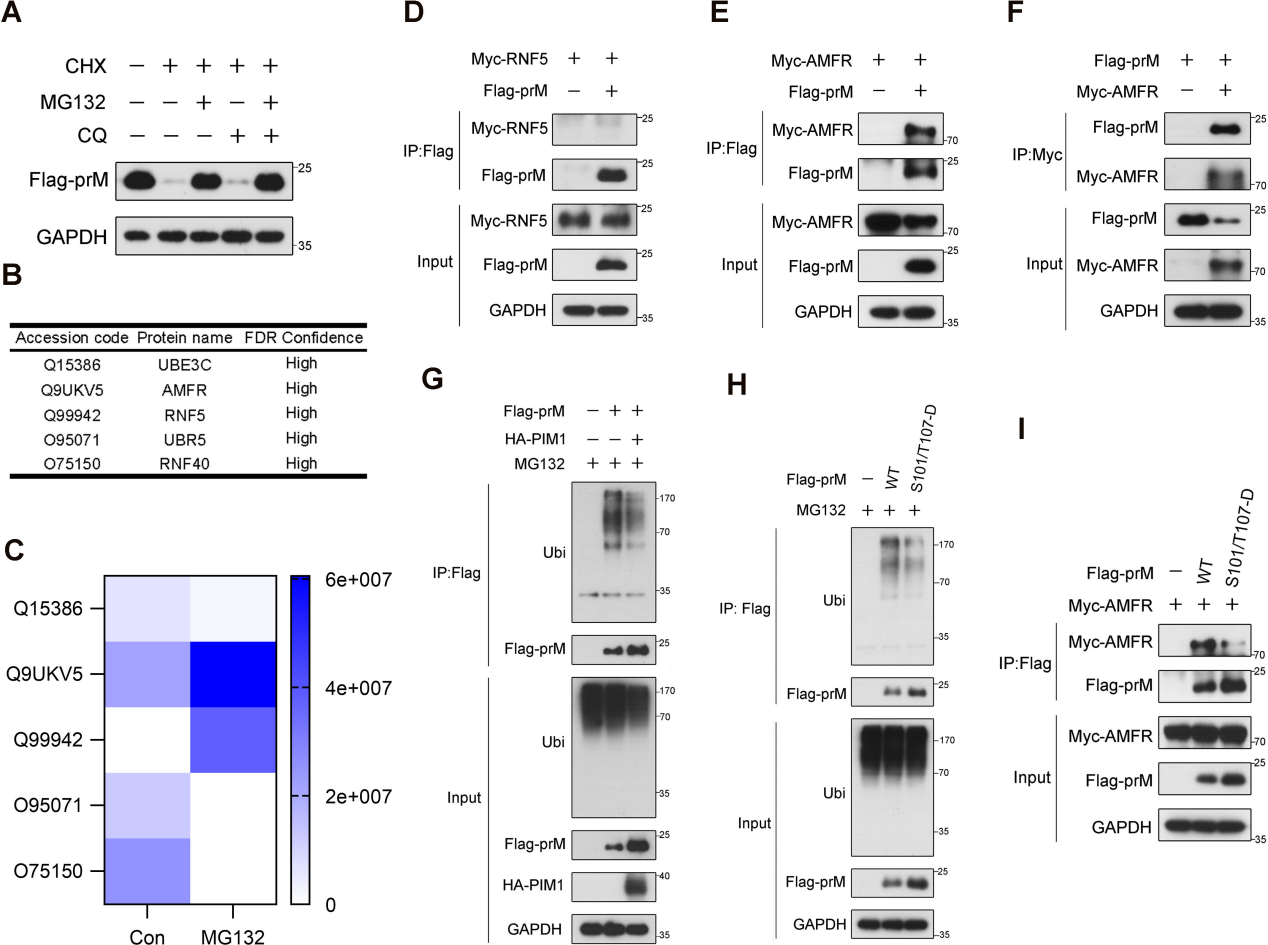
Inhibitory effect of prM phosphorylation on its ubiquitin-mediated degradation. **(A)** HEK293T cells were transfected with constructs expressing Flag-prM. At 24 h after transfection, the cells were treated with 50 μg/mL CHX with or without 10 μM MG132 or 50 μM CQ for 12 h before lysis. The cell lysates were analyzed by immunoblotting with the indicated antibodies. **(B)** The E3 ubiquitin ligases interacting with prM. HEK293T cells were transfected with constructs expressing Flag-prM. At 24 h after transfection, the cells were treated with 10 μM MG132 for 6 h before lysis. The cell lysates were subjected to immunoprecipitation with an anti-Flag antibody, and the bead-bound proteins were analyzed by MS/MS. **(C)** Thermograph of the change in the abundance of the E3 ubiquitin ligases interacting with prM. **(D, E)** HEK293T cells were transfected with constructs expressing Flag-prM and Myc-RNF5 **(D)** or Myc-AMFR **(E)** for 24 h. The cell lysates were subjected to immunoprecipitation with an anti-Flag antibody and analyzed by immunoblotting with the indicated antibodies. **(F)** HEK293T cells were transfected with constructs expressing Flag-prM and Myc-AMFR for 24 h. The cell lysates were subjected to immunoprecipitation with an anti-Myc antibody and analyzed by immunoblotting with the indicated antibodies. **(G)** HEK293T cells were transfected with the HA control or HA-PIM1 vector, together with the Flag control or Flag-prM vector. At 24 h after transfection, the cells were treated with 10 μM MG132 for 3 h before lysis. The cell lysates were subjected to immunoprecipitation with an anti-Flag antibody and analyzed by immunoblotting with the indicated antibodies. **(H)** HEK293T cells were transfected with the Flag control, Flag-prM vector or its phosphorylation mimic mutants. At 24 h after transfection, the cells were treated with 10 μM MG132 for 3 h before lysis. The cell lysates were subjected to immunoprecipitation with an anti-Flag antibody and analyzed by immunoblotting with the indicated antibodies. **(I)** HEK293T cells were transfected with the Myc-AMFR vector and the Flag control, Flag-prM or its phosphorylation mimic mutants for 24 h. The cell lysates were subjected to immunoprecipitation with an anti-Flag antibody and analyzed by immunoblotting with the indicated antibodies.

Given that PIM1 depends on its kinase activity to increase the half-life of prM, we hypothesized that PIM1 inhibits prM degradation to increase its stability. To verify this hypothesis, the primary proteolytic pathway of the prM protein was investigated by treating HEK293T cells transfected with constructs expressing Flag-prM with the proteasome inhibitor MG132 and the autophagy inhibitor chloroquine (CQ) in the presence of the eukaryotic protein synthesis inhibitor CHX. As shown in **Figure 6A**, treatment with MG132 restored the protein level of prM when CHX inhibited protein synthesis, whereas CQ had no effect on the protein level of prM, revealing that the prM protein is degraded mainly through the ubiquitin–proteasome pathway. We also validated this phenomenon in A549 cells, which are susceptible to ZIKV (**Supplementary Figure S8**). To further understand the mechanism underlying the proteasomal degradation of the prM protein, HEK293T cells transfected with constructs expressing Flag-prM were treated with MG132 to enrich the prM proteins that interact with the E3 ubiquitin ligase responsible for the proteasomal degradation of the prM protein, after which the proteins were immunoprecipitated via an anti-Flag antibody and analyzed by MS/MS. The analysis of the proteins immunoprecipitated by MS/MS revealed that the E3 ubiquitin ligases AMFR and RNF5 were up-regulated when proteasomal degradation was inhibited by MG132, indicating that AMFR and RNF5 may mediate the ubiquitination of prM (**Figures 6B, C**). To identify the exact E3 ubiquitin ligase, we performed co-immunoprecipitation in HEK293T cells co-transfected with constructs expressing Flag-prM and Myc-RNF5 or Myc-AMFR, and immunoblotting analysis revealed that AMFR interacted with prM, but not RNF5 (**Figures 6D, E**). We performed co-immunoprecipitation again using an anti-Myc antibody to validate the interaction between AMFR and prM (**Figure 6F**). These results indicate that the E3 ubiquitin ligase AMFR mediates the proteasomal degradation of the prM protein.

To further investigate the effect of PIM1 on prM degradation, a ubiquitination assay was performed to examine the amount of ubiquitin covalently bound to prM when PIM1 was overexpressed. Interestingly, immunoblotting analysis indicated that the overexpression of PIM1 extremely decreased the amount of ubiquitin bound to prM (**Figure 6G**). To further examine the relevance of PIM1 kinase activity to prM degradation, HEK293T cells were transfected with constructs expressing phosphorylation mimic mutants of Flag-prM, and the ubiquitination assay revealed that the ubiquitin amount of prM phosphorylation mimic mutants extremely decreased compared with that of wild-type prM (**Figure 6H**). Furthermore, we performed co-immunoprecipitation in HEK293T cells transfected with the Myc-AMFR vector and Flag control, Flag-prM or its phosphorylation mimic mutants. Immunoblotting analysis revealed that the protein level of AMFR bound to prM phosphorylation mimic mutants was lower than that of the wild-type prM, indicating that PIM1 phosphorylated prM to impair the interaction between AMFR and the prM protein, thereby preventing the degradation of prM (**Figure 6I**). In summary, these data demonstrate that PIM1 phosphorylates prM to decrease the ubiquitination of prM thereby preventing its degradation.

### 3.7 Inhibition of ZIKV by SGI-1776 in a type I IFN signaling-independent manner

**Figure 7:**
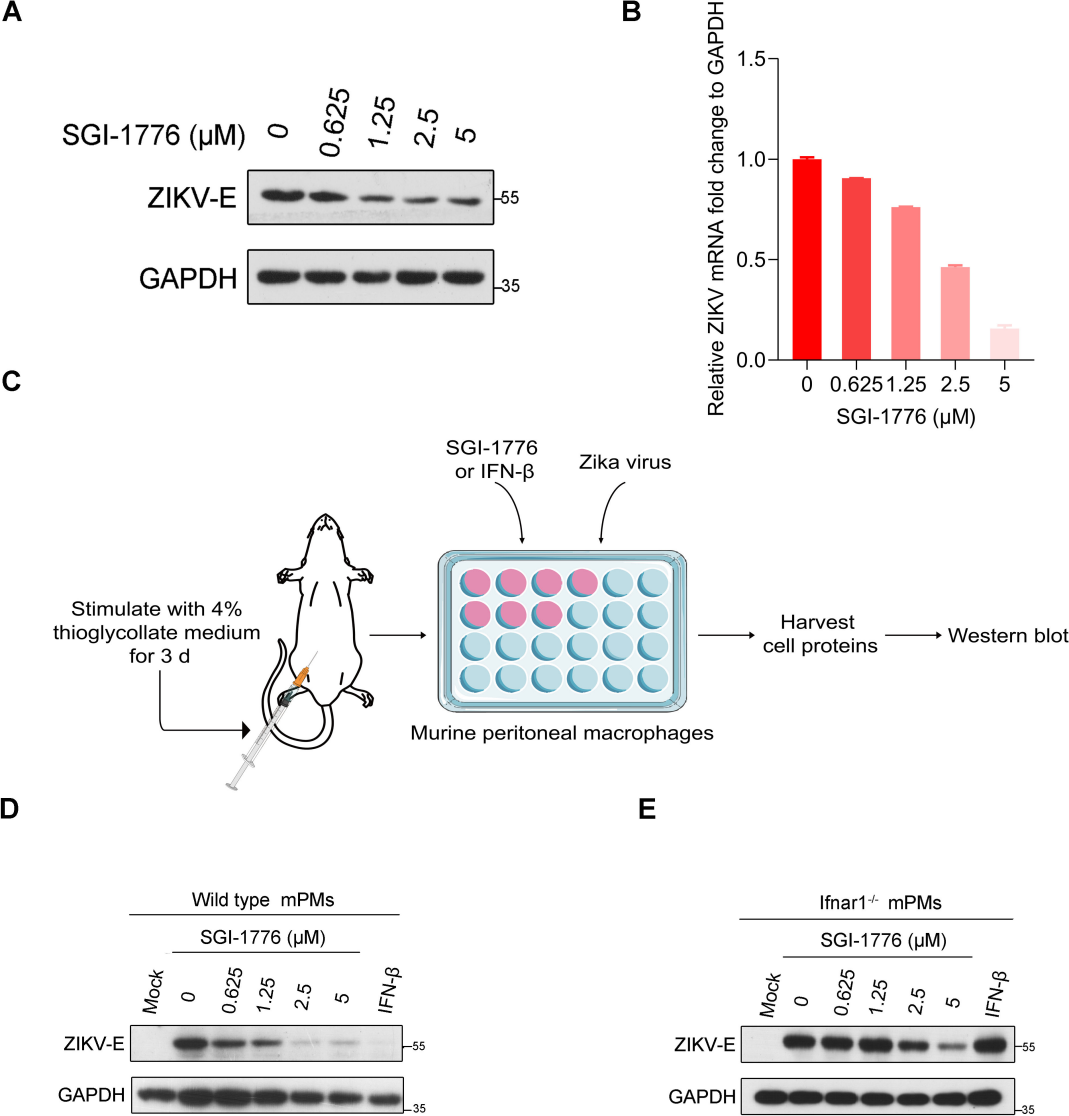
Inhibition of ZIKV by SGI-1776 in a type I IFN signaling independent manner. **(A)** A549 cells were infected with ZIKV at an MOI of 0.1 and incubated with the indicated concentration of SGI-1776 for 48 h. The cell lysates were analyzed by immunoblotting with the indicated antibodies. **(B)** A549 cells were infected with ZIKV at an MOI of 0.1 and incubated with the indicated concentration of SGI-1776 for 48 h. The cellular viral RNA level was determined by RT−qPCR. **(C)** Schematic illustration of the effect of SGI-1776 on ZIKV replication in murine peritoneal macrophages. **(D, E)** Murine peritoneal macrophages isolated from six-week-old wild-type **(D)** or *Ifnar1* ^-/-^ **(E)** C57BL/6 mice were infected with ZIKV at an MOI of 0.1 and incubated with the indicated concentrations of SGI-1776 or 10 ng/mL IFN-β for 48 h. The cell lysates were analyzed by immunoblotting with the indicated antibodies.

On the basis of the above results, we conclude that ZIKV hijacks PIM1 kinase for prM phosphorylation to prevent ubiquitin−mediated degradation and facilitate viral replication. The PIM1 kinase inhibitor SGI-1776 should be a specific antiviral agent for ZIKV. However, PIM1 was previously reported to promote Zika virus replication by inhibiting type I IFN signaling in host cells (Zhou et al., 2021). Thereafter, we wanted to determine whether SGI-1776 inhibits ZIKV in a type I IFN signaling-independent manner during ZIKV infection. When A549 cells infected with ZIKV at an MOI of 0.1 were treated with the PIM1 inhibitor SGI-1776, both the intracellular envelope protein level and the RNA level of ZIKV were markedly suppressed by SGI-1776 in a dose-dependent manner at 48 h post-infection (**Figures 7A, B**). And the prM protein level of ZIKV was also markedly suppressed by SGI-1776 in a dose-dependent manner at 48 h post-infection (**Supplementary Figure S9**). Besides, when mPMs isolated from six-week-old wild-type C57BL/6 mice were infected with ZIKV at an MOI of 0.1 and treated with the indicated concentrations of SGI-1776, as displayed in **Figure 7C**, we also observed that the intracellular envelope protein level of ZIKV was suppressed by SGI-1776 in a dose-dependent manner at 48 h post-infection (**Figure 7D**). Interestingly, when the source of mPMs was changed to six-week-old *Ifnar1^-/-^* C57BL/6 mice, SGI-1776 still suppressed the intracellular envelope protein level of ZIKV in a dose-dependent manner, revealing that SGI-1776 could inhibit ZIKV replication in a type I IFN signaling-independent manner (**Figure 7E**). Together, these findings indicate that PIM1 can promote ZIKV replication independently of the type Ι IFN signaling pathway.

## 4 Discussion

A previous report revealed that ZIKV infection can stimulate PIM1 kinase expression, and that PIM1 facilitates ZIKV replication by suppressing host cell type I IFN signaling activity (Zhou et al., 2021). As PIM1 directly interacts with the HCV NS5A protein to regulate HCV entry (Park et al., 2015), we performed a series of co-immunoprecipitation experiments in HEK293T cells transfected with the indicated constructs to determine whether ZIKV proteins interact with PIM1. We found that PIM1 could interact with structural protein prM and nonstructural protein NS1 (**Figures 1C, E**). Although ZIKV prM and NS1 were usually considered to locate in ER lumen based on the topology of ZIKV polyprotein, prM and NS1 were reported to directly interact with some cytosolic proteins (Xia et al., 2018; Zheng et al., 2018; Hui et al., 2020; Sui et al., 2023). Really, we validated the direct interaction between PIM1 and prM through *in vitro* GST pull-down (**Figure 2C**) and immunofluorescence staining (**Supplementary Figure S10**).

Apparently, the protein level of prM was markedly increased by PIM1 (**Figure 1C**, **Supplementary Figure S1A**). Owing to the pivotal role of the prM protein in the formation of ZIKV infectious virions (Stadler et al., 1997; Yu et al., 2009; Sirohi and Kuhn, 2017; Imran et al., 2019; Li et al., 2019; Gwon et al., 2020; Renner et al., 2021; Goellner et al., 2023) and the short half-life of the prM protein shown in **Figure 4C**, which is less than 30 minutes, PIM1 likely plays an important role in the formation of ZIKV infectious virions. In addition, the expression level of PIM1 is high in placenta and brain tissue, which is in accordance with the ZIKV tropism for placenta and neural tissue (Miner and Diamond, 2017; Shaily and Upadhya, 2019). Thus, we speculated that the high expression level of PIM1 may have an important effect on fetal microcephaly caused by ZIKV infection in pregnant women.

Many reports have revealed that protein kinases play important roles in modulating the life cycle of viruses. For example, receptor-interacting serine/threonine-protein kinase 1 (RIPK1), cyclin-dependent kinase 2 (CDK2), P21-activated kinase 1 (PAK1), adenosine monophosphate-activated protein kinase (AMPK) and its related kinase novel (nua) kinase (NUAK)-2 are hijacked by SARS-CoV-2 to facilitate its entry or replication (Xu et al., 2021; Guo et al., 2022; Liu et al., 2023; Prasad et al., 2023). Protein kinase A (PKA) and tyrosine kinase c-Abl1 are hijacked by the Ebola virus to facilitate its replication (García et al., 2012; Zhu et al., 2022). DNA-dependent protein kinase (DNA-PK) and AKT serine/threonine kinases are hijacked by HIV to promote its replication or infectivity (Zicari et al., 2020; Raja et al., 2022). For ZIKV, the tyrosine-protein kinase Lyn and the mechanistic (mammalian) target of rapamycin (mTOR) are hijacked to promote its egress and replication (Li et al., 2020; Sahoo et al., 2020). Therefore, we performed a variety of experiments to study how the direct interaction between PIM1 and prM affects the life cycle of ZIKV.

Because of the direct interaction between PIM1 and the prM protein, we hypothesized that kinase activity is related to the effect of PIM1 on the up-regulation of prM. Similarly, we found that the effect of PIM1 on the up-regulation of prM occurs in the post-translational stage and that the kinase activity of PIM1 is indispensable for the up-regulation of prM stability (**Figure 4**). Above all, we found that PIM1 directly phosphorylates prM through an *in vitro* kinase assay (**Figure 5A**). These findings suggest that the protein kinase PIM1 phosphorylates the ZIKV structural protein prM to increase its cellular abundance, which is of benefit to ZIKV replication. There had a report that PIM1 directly phosphorylated the accessary protein Vpx encoded by human immunodeficiency virus type 2 (HIV-2) and some strains of simian immunodeficiency virus (SIV) to stabilize the interaction of Vpx with an the intrinsic host restriction factor sterile alpha motif and histidine-aspartate domain-containing protein 1 (SAMHD1), which was identified as an inhibitor of several lentiviruses, thereby promoting ubiquitin−mediated proteolysis of SAMHD1 to facilitate lentiviral evasion (Hrecka et al., 2011; Miyakawa et al., 2019). However, the facilitation effect on the lentiviral evasion was by affecting the stability of the host cell protein SAMHD1.

Moreover, we also found that the prM protein is degraded mainly through the ubiquitin−proteasome pathway and identified that the E3 ubiquitin ligase AMFR is responsible for the ubiquitination of the prM protein (**Figures 6A–F**). Some reports have described the relationship between protein kinases and the ubiquitin−mediated proteolysis of their substrates. For example, apoptosis signal-regulating kinase 1 (ASK1) phosphorylates histone deacetylase 6 (HDAC6) and inhibits the ubiquitin−mediated proteolysis of HDAC6, which contributes to the pathology induced by oxygen changes, suggesting a potential target for the treatment of retinopathy of prematurity (Ran et al., 2020). In contrast, the nonreceptor tyrosine kinase Fyn-related kinase (FRK) phosphorylates yes-associated protein (YAP) and induces its ubiquitin−mediated proteolysis, contributing to the inhibition of glioblastoma progression (Wang et al., 2022). PIM1 directly phosphorylates HIF-1α and inhibits its ubiquitin−mediated degradation through the disruption of the hydroxylation of HIF-1α by prolyl hydroxylases, driving angiogenesis in solid tumors (Casillas et al., 2021). PIM1 phosphorylates S-phase kinase-associated protein 2 (Skp2) and increases its stability by inhibiting its ubiquitin−mediated degradation, promoting the degradation of p27, a critical regulator of cyclin-dependent kinases that mediate cell cycle progression (Cen et al., 2010). Therefore, we investigated whether PIM1 affects the ubiquitination of the prM protein, and found that the overexpression of PIM1 decreased the ubiquitination of the prM protein (**Figure 6G**). Furthermore, compared with that of wild-type prM, the degree of ubiquitination of prM phosphorylation mimic mutants extremely decreased (**Figure 6H**), indicating that the phosphorylation of prM catalyzed by PIM1 prevents the ubiquitin−mediated degradation of prM, thereby increasing the stability of prM. We also found that the phosphorylation of prM impaired the interaction between AMFR and the prM protein, explaining how the phosphorylation effect of PIM1 on the prM protein affects the ubiquitination of prM (**Figure 6I**). In summary, besides the wide range of host cell proteins, our study discovered a virus protein degraded by the ubiquitin−proteasome pathway as a new PIM1 substrate, which has enriched the research on the mechanisms of the cross-talk between the phosphorylation and the ubiquitination of proteins.

Previous studies reported that the upregulation of PIM1 could decrease the phosphorylation of the STAT1/STAT2 complex, a transcription activator of interferon-stimulated genes (ISGs) involved in antiviral activity, thereby suppressing the type I IFN signaling pathway to facilitate ZIKV replication (Zhou et al., 2021). We also found that SGI-1776, a PIM kinase inhibitor, could suppress ZIKV replication in a dose-dependent manner in A549 cells and the mPMs isolated from wild-type C57BL/6 mice with a complete type I IFN signaling pathway (**Figures 7A, B, D**). Interestingly, SGI-1776 could still suppress ZIKV replication in the primary mPMs in which the type I IFN receptor was knocked out, revealing that SGI-1776 could inhibit ZIKV replication in a type I IFN signaling-independent manner, indicating that PIM1 could facilitate ZIKV replication by directly phosphorylating the ZIKV structural protein prM to prevent the ubiquitin−mediated proteolysis of prM so as to promote the stability of prM, thereby facilitating ZIKV replication (**Figure 7E**). In this study, we revealed a new mechanism by which PIM1 facilitates ZIKV replication through direct phosphorylation on prM, supplementing the findings of a previous study showing that PIM1 facilitates ZIKV replication by suppressing the type I IFN signaling pathway.

In summary, our study revealed PIM1 as a host factor that benefits ZIKV replication by directly phosphorylating the virus protein prM to prevent its ubiquitin−proteasome-mediated degradation. These findings highlight the importance of PIM1 inhibition as a therapeutic target and interventional strategy for antiviral therapy.

## Data Availability

The raw data supporting the conclusions of this article will be made available by the authors, without undue reservation.

## References

[B1] Bachmann M. Möröy T. (2005). The serine/threonine kinase Pim-1. Int. J. Biochem. Cell Biol. 37, 726–730. doi: 10.1016/j.biocel.2004.11.005 15694833

[B2] Blanco-Aparicio C. Carnero A. (2013). Pim kinases in cancer: diagnostic, prognostic and treatment opportunities. Biochem. Pharmacol. 85, 629–643. doi: 10.1016/j.bcp.2012.09.018 23041228

[B3] Casillas A. L. Chauhan S. S. Toth R. K. Sainz A. G. Clements A. N. Jensen C. C.. (2021). Direct phosphorylation and stabilization of HIF-1α by PIM1 kinase drives angiogenesis in solid tumors. Oncogene. 40, 5142–5152. doi: 10.1038/s41388-021-01915-1 34211090 PMC8364516

[B4] Cen B. Mahajan S. Zemskova M. Beharry Z. Lin Y. W. Cramer S. D.. (2010). Regulation of Skp2 levels by the Pim-1 protein kinase. J. Biol. Chem. 285, 29128–29137. doi: 10.1074/jbc.M110.137240 20663873 PMC2937943

[B5] Cuypers H. T. Selten G. Quint W. Zijlstra M. Maandag E. R. Boelens W.. (1984). Murine leukemia virus-induced T-cell lymphomagenesis: integration of proviruses in a distinct chromosomal region. Cell. 37, 141–150. doi: 10.1016/0092-8674(84)90309-X 6327049

[B6] de Vries M. Smithers N. P. Howarth P. H. Nawijn M. C. Davies D. E. (2015). Inhibition of Pim1 kinase reduces viral replication in primary bronchial epithelial cells. Eur. Respir. J. 45, 1745–1748. doi: 10.1183/09031936.00206514 25745039

[B7] García M. Cooper A. Shi W. Bornmann W. Carrion R. Kalman D.. (2012). Productive replication of Ebola virus is regulated by the c-Abl1 tyrosine kinase. Sci. Trans. Med. 4, 123ra24. doi: 10.1126/scitranslmed.3003500 PMC4794994 22378924

[B8] Giovannoni F. Bosch I. Polonio C. M. Torti M. F. Wheeler M. A. Li Z.. (2020). AHR is a Zika virus host factor and a candidate target for antiviral therapy. Nat. Neurosci. 23, 939–951. doi: 10.1038/s41593-020-0664-0 32690969 PMC7897397

[B9] Goellner S. Enkavi G. Prasad V. Denolly S. Eu S. Mizzon G.. (2023). Zika virus prM protein contains cholesterol binding motifs required for virus entry and assembly. Nat. Commun. 14, 7344. doi: 10.1038/s41467-023-42985-x 37957166 PMC10643666

[B10] Guo S. Lei X. Chang Y. Zhao J. Wang J. Dong X.. (2022). SARS-CoV-2 hijacks cellular kinase CDK2 to promote viral RNA synthesis. Signal Transduct Target Ther. 7, 400. doi: 10.1038/s41392-022-01239-w 36575184 PMC9793359

[B11] Gwon Y. D. Zusinaite E. Merits A. Överby A. K. Evander M. (2020). N-glycosylation in the pre-membrane protein is essential for the zika virus life cycle. Viruses 12, 925. doi: 10.3390/v12090925 32842538 PMC7552079

[B12] Hamel R. Dejarnac O. Wichit S. Ekchariyawat P. Neyret A. Luplertlop N.. (2015). Biology of zika virus infection in human skin cells. J. Virol. 89, 8880–8896. doi: 10.1128/JVI.00354-15 26085147 PMC4524089

[B13] Hrecka K. Hao C. Gierszewska M. Swanson S. K. Kesik-Brodacka M. Srivastava S.. (2011). Vpx relieves inhibition of HIV-1 infection of macrophages mediated by the SAMHD1 protein. Nature. 474, 658–661. doi: 10.1038/nature10195 21720370 PMC3179858

[B14] Hui L. Nie Y. Li S. Guo M. Yang W. Huang R.. (2020). Matrix metalloproteinase 9 facilitates Zika virus invasion of the testis by modulating the integrity of the blood-testis barrier. PLoS pathogens. 16, e1008509. doi: 10.1371/journal.ppat.1008509 32302362 PMC7190178

[B15] Imran M. Saleemi M. K. Chen Z. Wang X. Zhou D. Li Y.. (2019). Decanoyl-Arg-Val-Lys-Arg-Chloromethylketone: An Antiviral Compound That Acts against Flaviviruses through the Inhibition of Furin-Mediated prM Cleavage. Viruses 11, 1011. doi: 10.3390/v11111011 31683742 PMC6893617

[B16] Inagaki T. Taniguchi S. Kawai Y. Maeki T. Nakayama E. Tajima S.. (2021). Leu-to-Phe substitution at prM(146) decreases the growth ability of Zika virus and partially reduces its pathogenicity in mice. Sci. Rep. 11, 19635. doi: 10.1038/s41598-021-99086-2 34608212 PMC8490429

[B17] Ko R. Seo J. Park H. Lee N. Lee S. Y. (2022). Pim1 promotes IFN-β production by interacting with IRF3. Exp. Mol. Med. 54, 2092–2103. doi: 10.1038/s12276-022-00893-y 36446848 PMC9722908

[B18] Kumar A. Mandiyan V. Suzuki Y. Zhang C. Rice J. Tsai J.. (2005). Crystal structures of proto-oncogene kinase Pim1: a target of aberrant somatic hypermutations in diffuse large cell lymphoma. J. Mol. Biol. 348, 183–193. doi: 10.1016/j.jmb.2005.02.039 15808862

[B19] Li G. Bos S. Tsetsarkin K. A. Pletnev A. G. Desprès P. Gadea G.. (2019). The roles of prM-E proteins in historical and epidemic zika virus-mediated infection and neurocytotoxicity. Viruses 11, 157. doi: 10.3390/v11020157 30769824 PMC6409645

[B20] Li M. Y. Naik T. S. Siu L. Y. L. Acuto O. Spooner E. Wang P.. (2020). Lyn kinase regulates egress of flaviviruses in autophagosome-derived organelles. Nat. Commun. 11, 5189. doi: 10.1038/s41467-020-19028-w 33060596 PMC7564011

[B21] Liu M. Lu B. Li Y. Yuan S. Zhuang Z. Li G.. (2023). P21-activated kinase 1 (PAK1)-mediated cytoskeleton rearrangement promotes SARS-CoV-2 entry and ACE2 autophagic degradation. Signal Transduct Target Ther. 8, 385. doi: 10.1038/s41392-023-01631-0 37806990 PMC10560660

[B22] Ma J. Arnold H. K. Lilly M. B. Sears R. C. Kraft A. S. (2007). Negative regulation of Pim-1 protein kinase levels by the B56beta subunit of PP2A. Oncogene. 26, 5145–5153. doi: 10.1038/sj.onc.1210323 17297438

[B23] Maslow J. N. Roberts C. C. (2020). Zika virus: A brief history and review of its pathogenesis rediscovered. Methods Mol. Biol. (Clifton NJ). 2142, 1–8. doi: 10.1007/978-1-0716-0581-3_1 32367354

[B24] Miner J. J. Diamond M. S. (2017). Zika virus pathogenesis and tissue tropism. Cell Host Microbe 21, 134–142. doi: 10.1016/j.chom.2017.01.004 28182948 PMC5328190

[B25] Miyakawa K. Matsunaga S. Yokoyama M. Nomaguchi M. Kimura Y. Nishi M.. (2019). PIM kinases facilitate lentiviral evasion from SAMHD1 restriction via Vpx phosphorylation. Nat. Commun. 10, 1844. doi: 10.1038/s41467-019-09867-7 31015445 PMC6479052

[B26] Nawijn M. C. Alendar A. Berns A. (2011). For better or for worse: the role of Pim oncogenes in tumorigenesis. Nat. Rev. Cancer. 11, 23–34. doi: 10.1038/nrc2986 21150935

[B27] Park C. Min S. Park E. M. Lim Y. S. Kang S. Suzuki T.. (2015). Pim kinase interacts with nonstructural 5A protein and regulates hepatitis C virus entry. J. Virol. 89, 10073–10086. doi: 10.1128/JVI.01707-15 26202252 PMC4577879

[B28] Polier S. Samant R. S. Clarke P. A. Workman P. Prodromou C. Pearl L. H. (2013). ATP-competitive inhibitors block protein kinase recruitment to the Hsp90-Cdc37 system. Nat. Chem. Biol. 9, 307–312. doi: 10.1038/nchembio.1212 23502424 PMC5695660

[B29] Postler T. S. Beer M. Blitvich B. J. Bukh J. de Lamballerie X. Drexler J. F.. (2023). Renaming of the genus Flavivirus to Orthoflavivirus and extension of binomial species names within the family Flaviviridae. Arch. virology. 168, 224. doi: 10.1007/s00705-023-05835-1 37561168

[B30] Prasad V. Cerikan B. Stahl Y. Kopp K. Magg V. Acosta-Rivero N.. (2023). Enhanced SARS-CoV-2 entry via UPR-dependent AMPK-related kinase NUAK2. Mol. Cell. 83, 2559–77.e8. doi: 10.1016/j.molcel.2023.06.020 37421942

[B31] Qian K. C. Wang L. Hickey E. R. Studts J. Barringer K. Peng C.. (2005). Structural basis of constitutive activity and a unique nucleotide binding mode of human Pim-1 kinase. J. Biol. Chem. 280, 6130–6137. doi: 10.1074/jbc.M409123200 15525646

[B32] Raja R. Wang C. Mishra R. Das A. Ali A. Banerjea A. C. (2022). Host AKT-mediated phosphorylation of HIV-1 accessory protein Vif potentiates infectivity via enhanced degradation of the restriction factor APOBEC3G. J. Biol. Chem. 298, 101805. doi: 10.1016/j.jbc.2022.101805 35259395 PMC8980627

[B33] Ran J. Liu M. Feng J. Li H. Ma H. Song T.. (2020). ASK1-mediated phosphorylation blocks HDAC6 ubiquitination and degradation to drive the disassembly of photoreceptor connecting cilia. Dev. Cell. 53, 287–99.e5. doi: 10.1016/j.devcel.2020.03.010 32275885

[B34] Renner M. Dejnirattisai W. Carrique L. Martin I. S. Karia D. Ilca S. L.. (2021). Flavivirus maturation leads to the formation of an occupied lipid pocket in the surface glycoproteins. Nat. Commun. 12, 1238. doi: 10.1038/s41467-021-21505-9 33623019 PMC7902656

[B35] Sahoo B. R. Pattnaik A. Annamalai A. S. Franco R. Pattnaik A. K. (2020). Mechanistic target of rapamycin signaling activation antagonizes autophagy to facilitate zika virus replication. J. Virol. 94, e01575–20. doi: 10.1128/JVI.01575-20 PMC7592218 32878890

[B36] Shaily S. Upadhya A. (2019). Zika virus: Molecular responses and tissue tropism in the mammalian host. Rev. Med. virology. 29, e2050. doi: 10.1002/rmv.v29.4 31095819

[B37] Sirohi D. Chen Z. Sun L. Klose T. Pierson T. C. Rossmann M. G.. (2016). The 3. 8 Å resolution cryo-EM structure Zika virus. Sci. (New York NY) 352, 467–470. doi: 10.1126/science.aaf5316 PMC4845755 27033547

[B38] Sirohi D. Kuhn R. J. (2017). Zika virus structure, maturation, and receptors. J. Infect. Dis. 216, S935–Ss44. doi: 10.1093/infdis/jix515 29267925 PMC5853281

[B39] Stadler K. Allison S. L. Schalich J. Heinz F. X. (1997). Proteolytic activation of tick-borne encephalitis virus by furin. J. Virol. 71, 8475–8481. doi: 10.1128/jvi.71.11.8475-8481.1997 9343204 PMC192310

[B40] Sui L. Zhao Y. Wang W. Chi H. Tian T. Wu P.. (2023). Flavivirus prM interacts with MDA5 and MAVS to inhibit RLR antiviral signaling. Cell bioscience. 13, 9. doi: 10.1186/s13578-023-00957-0 36639652 PMC9837762

[B41] Taniguchi H. Hasegawa H. Sasaki D. Ando K. Sawayama Y. Imanishi D.. (2014). Heat shock protein 90 inhibitor NVP-AUY922 exerts potent activity against adult T-cell leukemia-lymphoma cells. Cancer science. 105, 1601–1608. doi: 10.1111/cas.2014.105.issue-12 25263741 PMC4317953

[B42] Wang Z. Bhattacharya N. Weaver M. Petersen K. Meyer M. Gapter L.. (2001). Pim-1: a serine/threonine kinase with a role in cell survival, proliferation, differentiation and tumorigenesis. J. veterinary science. 2, 167–179. doi: 10.4142/jvs.2001.2.3.167 12441685

[B43] Wang Y. Wang K. Fu J. Zhang Y. Mao Y. Wang X.. (2022). FRK inhibits glioblastoma progression via phosphorylating YAP and inducing its ubiquitylation and degradation by Siah1. Neuro-oncology. 24, 2107–2120. doi: 10.1093/neuonc/noac156 35723276 PMC9713521

[B44] Xia H. Luo H. Shan C. Muruato A. E. Nunes B. T. D. Medeiros D. B. A.. (2018). An evolutionary NS1 mutation enhances Zika virus evasion of host interferon induction. Nat. Commun. 9, 414. doi: 10.1038/s41467-017-02816-2 29379028 PMC5788864

[B45] Xu G. Li Y. Zhang S. Peng H. Wang Y. Li D.. (2021). SARS-CoV-2 promotes RIPK1 activation to facilitate viral propagation. Cell Res. 31, 1230–1243. doi: 10.1038/s41422-021-00578-7 34663909 PMC8522117

[B46] Yang J. Liu K. Yang J. Jin B. Chen H. Zhan X.. (2017). PIM1 induces cellular senescence through phosphorylation of UHRF1 at Ser311. Oncogene. 36, 4828–4842. doi: 10.1038/onc.2017.96 28394343

[B47] Yu I. M. Holdaway H. A. Chipman P. R. Kuhn R. J. Rossmann M. G. Chen J. (2009). Association of the pr peptides with dengue virus at acidic pH blocks membrane fusion. J. Virol. 83, 12101–12107. doi: 10.1128/JVI.01637-09 19759134 PMC2786737

[B48] Yuan L. Huang X. Y. Liu Z. Y. Zhang F. Zhu X. L. Yu J. Y.. (2017). A single mutation in the prM protein of Zika virus contributes to fetal microcephaly. Sci. (New York NY). 358, 933–936. doi: 10.1126/science.aam7120 28971967

[B49] Zhang X. Yang W. Wang X. Zhang X. Tian H. Deng H.. (2018). Identification of new type I interferon-stimulated genes and investigation of their involvement in IFN-β activation. Protein Cell. 9, 799–807. doi: 10.1007/s13238-018-0511-1 29427062 PMC6107486

[B50] Zheng Y. Liu Q. Wu Y. Ma L. Zhang Z. Liu T.. (2018). Zika virus elicits inflammation to evade antiviral response by cleaving cGAS via NS1-caspase-1 axis. EMBO J. 37, e99347. doi: 10.15252/embj.201899347 30065070 PMC6138430

[B51] Zhou F. Wan Q. Chen Y. Chen S. He M. L. (2021). PIM1 kinase facilitates Zika virus replication by suppressing host cells' natural immunity. Signal Transduct Target Ther. 6, 207. doi: 10.1038/s41392-021-00539-x 34075019 PMC8169747

[B52] Zhou F. Wan Q. Lu J. Chen Y. Lu G. He M. L. (2019). Pim1 impacts enterovirus A71 replication and represents a potential target in antiviral therapy. iScience. 19, 715–727. doi: 10.1016/j.isci.2019.08.008 31476618 PMC6726883

[B53] Zhu L. Gao T. Huang Y. Jin J. Wang D. Zhang L.. (2022). Ebola virus VP35 hijacks the PKA-CREB1 pathway for replication and pathogenesis by AKIP1 association. Nat. Commun. 13, 2256. doi: 10.1038/s41467-022-29948-4 35474062 PMC9042921

[B54] Zicari S. Sharma A. L. Sahu G. Dubrovsky L. Sun L. Yue H.. (2020). DNA dependent protein kinase (DNA-PK) enhances HIV transcription by promoting RNA polymerase II activity and recruitment of transcription machinery at HIV LTR. Oncotarget. 11, 699–726. doi: 10.18632/oncotarget.27487 32133046 PMC7041937

