# Supplementary material for: ZIKV prM hijacks PIM1 kinase for phosphorylation to prevent ubiquitin−mediated degradation and facilitate viral replication
Source: Front Cell Infect Microbiol. 2024 Nov 29;14:1502770. doi: 10.3389/fcimb.2024.1502770 (PMC11638163; doi:10.3389/fcimb.2024.1502770)
Supplement: Supplementary file 1 [file DataSheet1.docx]

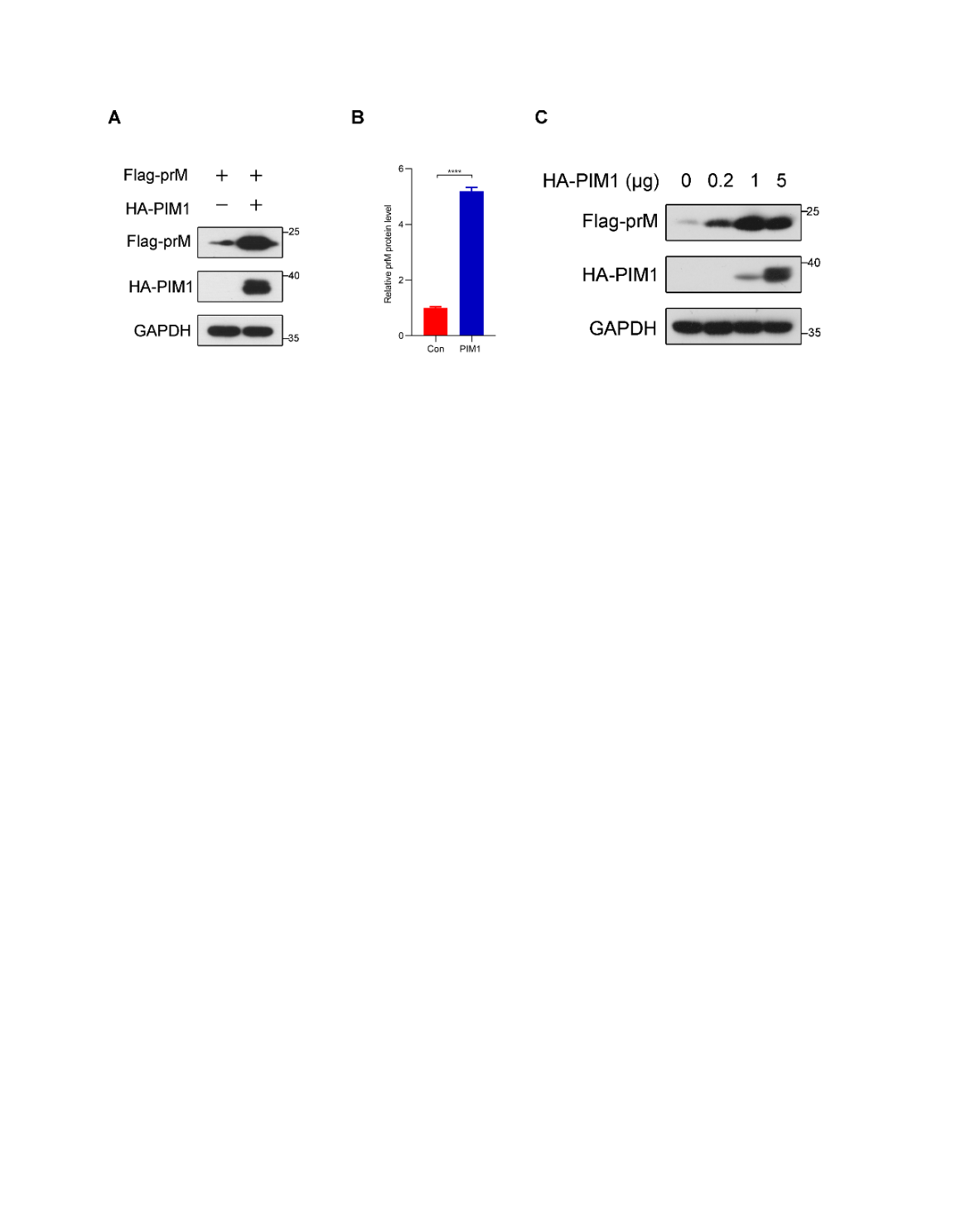


**Supplementary Figure 1.** Promotion effect of PIM1 on prM cellular abundance. (**A**) HEK293T cells were transfected with the HA control or HA-PIM1 vector, together with the Flag-prM vector for 24 h. The cell lysates were analyzed by immunoblotting with the indicated antibodies. (**B**) Quantification of relative prM protein levels in (A) (n = 3 independent experiments). The data are presented as the means ± SD. ****, *P* < 0.0001. Statistical analysis was performed with a two-tailed unpaired Student’s *t* test. (**C**) HEK293T cells were transfected with constructs expressing Flag-prM and HA-PIM1 with the indicated micrograms for 24 h. The cell lysates were analyzed by immunoblotting with the indicated antibodies.

**
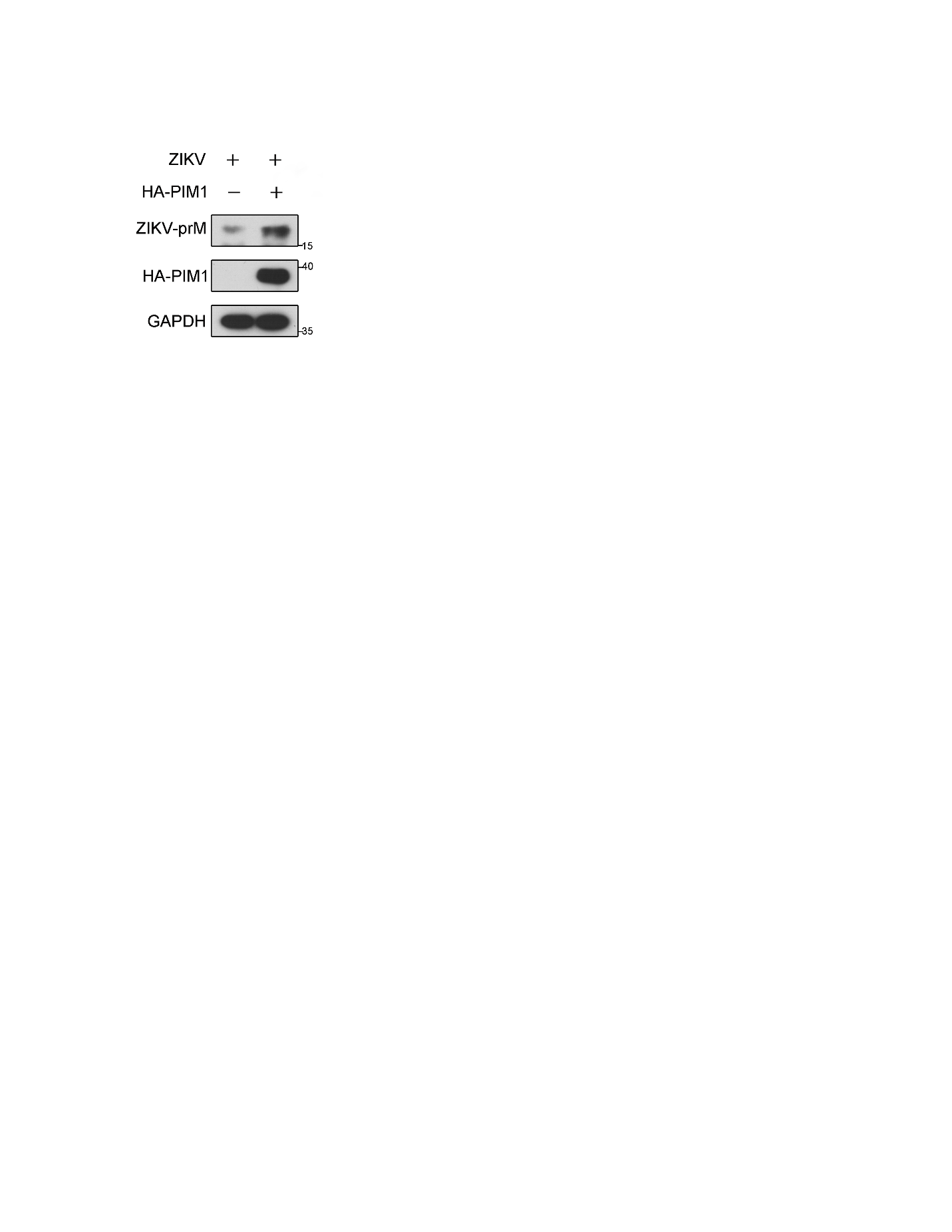
**

**Supplementary Figure 2.** Promotion effect of PIM1 on prM protein level under ZIKV infection. A549 cells were transfected with constructs expressing HA control or HA-PIM1. At 24 h after transfection, the cells were infected with ZIKV at an MOI of 0.1. The cell lysates were harvested and subjected to immunoblotting with indicated antibodies at 24 h post infection.


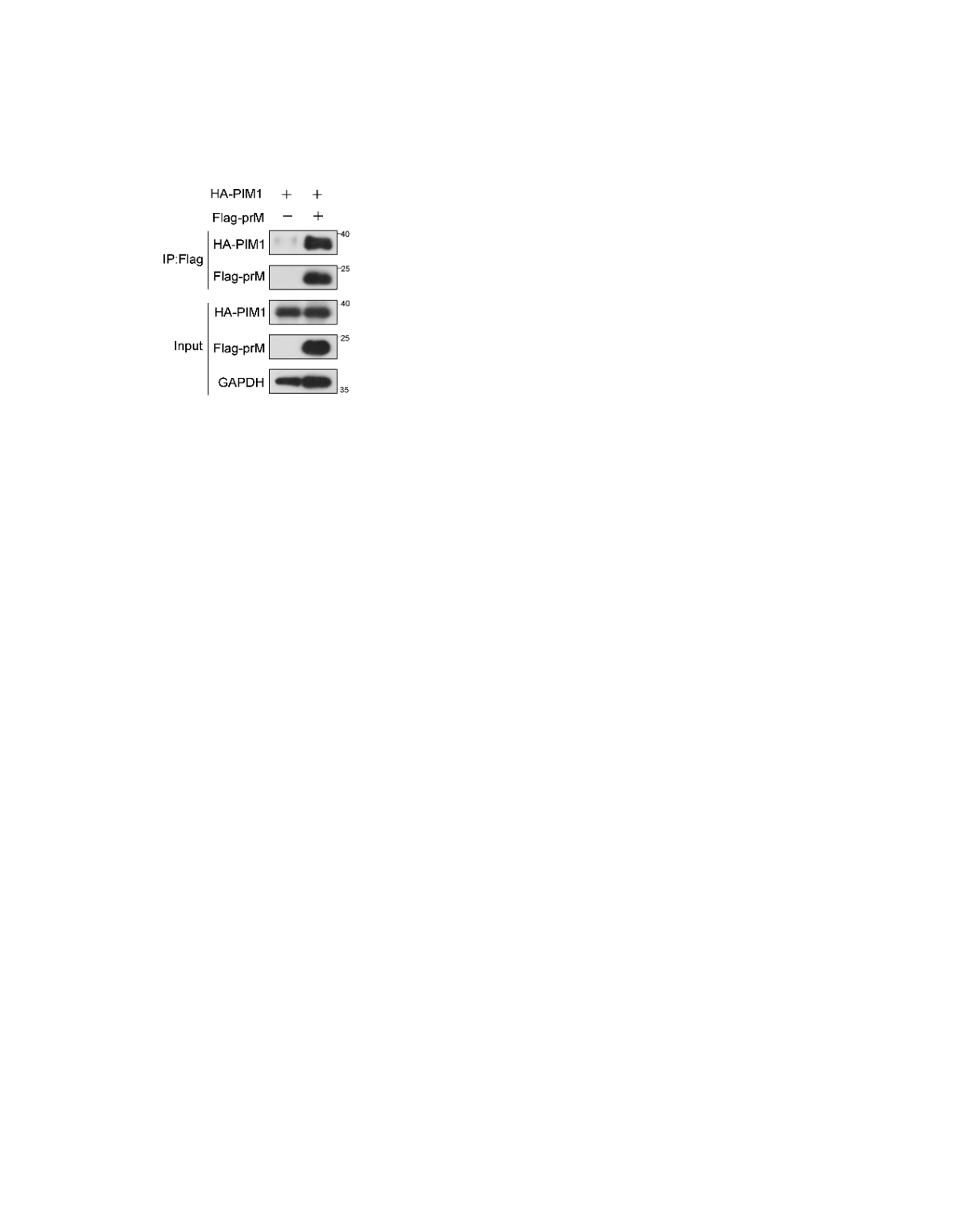


**Supplementary Figure 3.** Interaction between PIM1 and prM. HEK293T cells were transfected with constructs expressing HA-PIM1 and Flag-prM for 24 h. The cell lysates were subjected to immunoprecipitation with an anti-Flag antibody and analyzed by immunoblotting with the indicated antibodies.


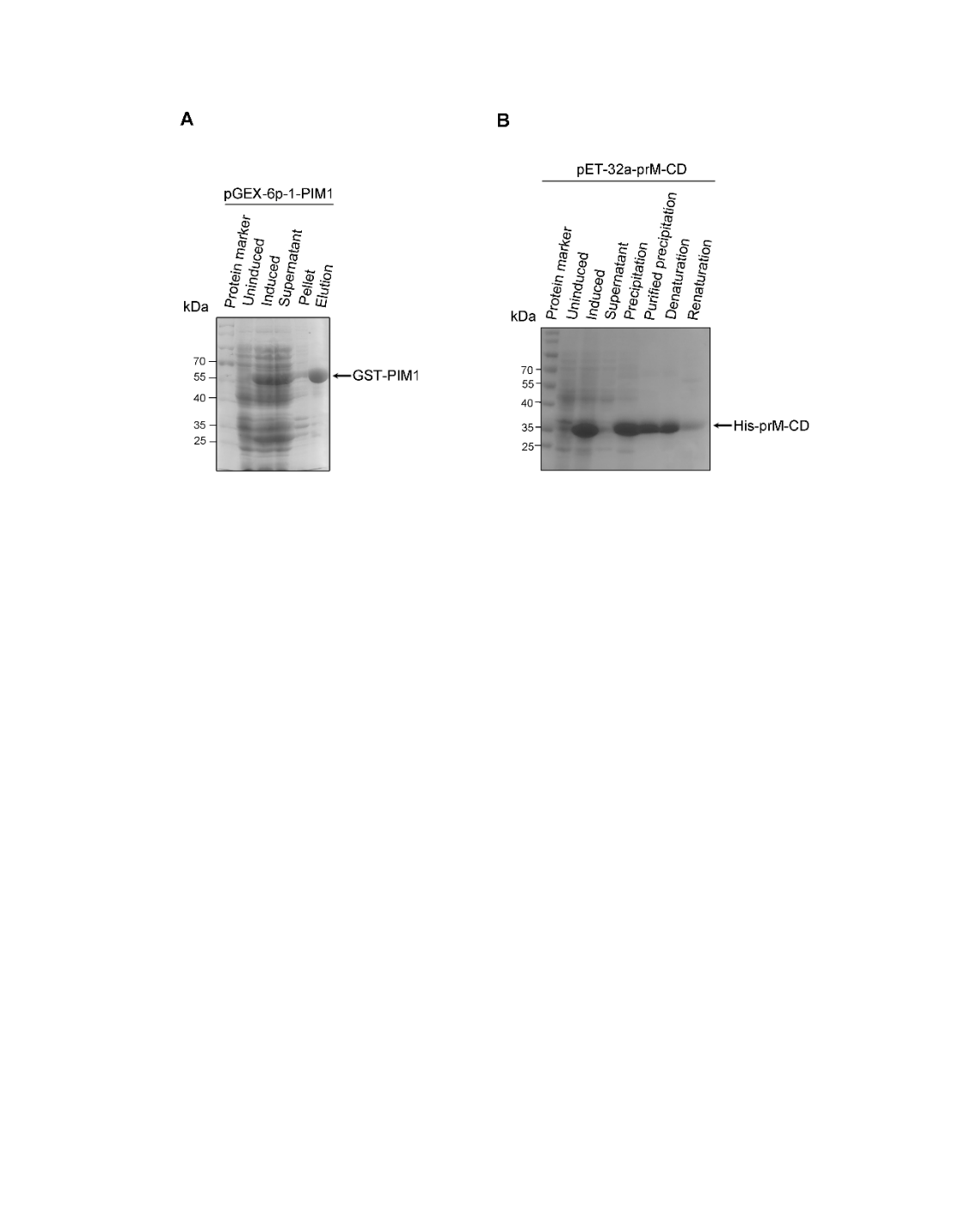


**Supplementary Figure 4.** Identification of recombinant human PIM1 and ZIKV prM via SDS‒PAGE. Figure illustrates the production and purification of recombinant human PIM1 (A) and ZIKV prM (B). The constructs expressing GST-PIM1 or 6×His-prM-CD were transformed into *Escherichia coli* Rosetta (DE3) competent cells and induced at the indicated temperature for the indicated time. The recombinant protein GST-PIM1 was purified via glutathione agarose resin (A). The inclusion body containing the recombinant protein 6×His-prM-CD was subjected to denaturation, purification with Ni-IDA resin, and renaturation to obtain the target recombinant protein (B). The indicated fractions were harvested and identified via Coomassie brilliant blue staining.


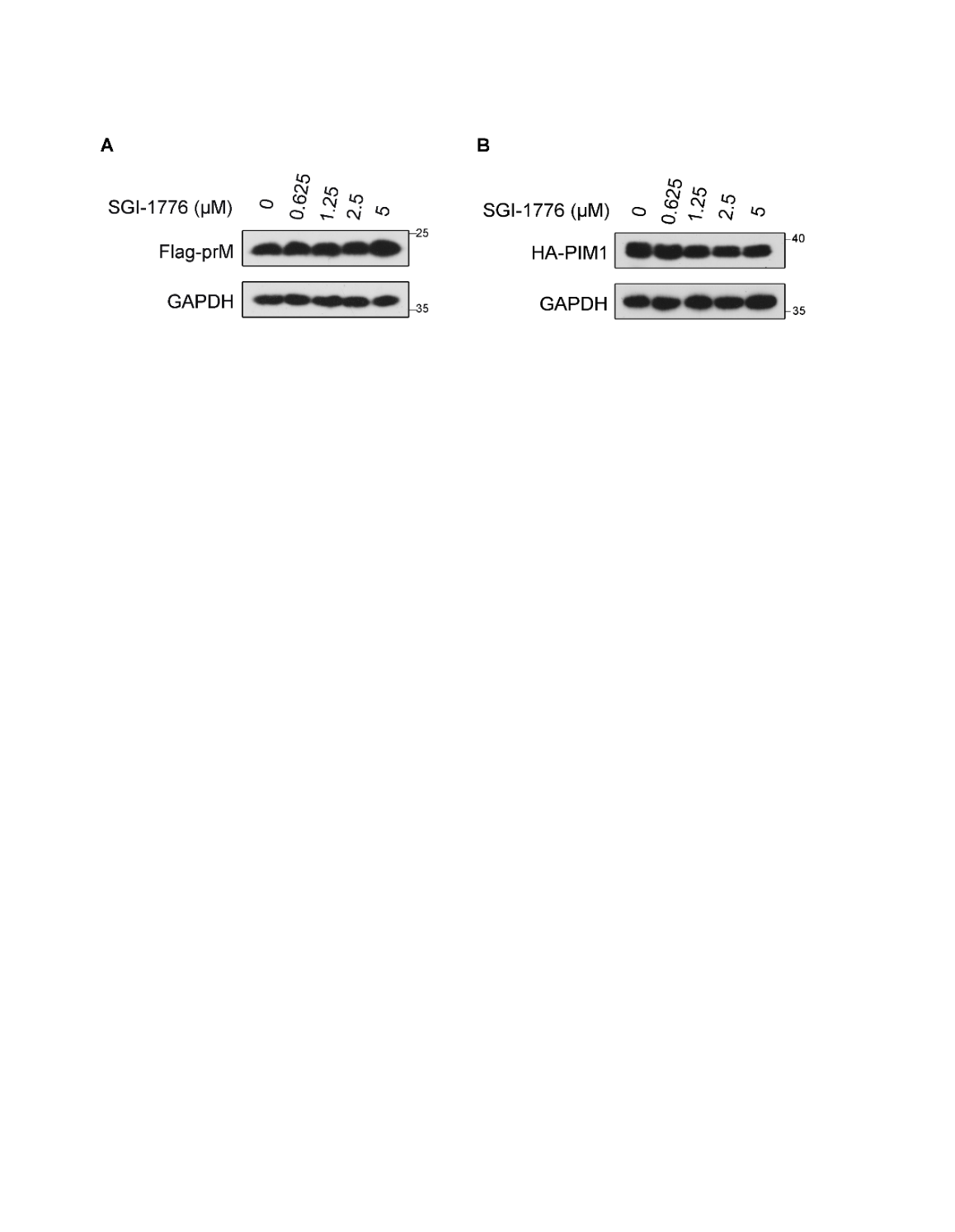


**Supplementary Figure 5.** Effects of SGI-1776 on prM and PIM1 cellular abundances. HEK293T cells were transfected with constructs expressing Flag-prM (A) or HA-PIM1 (B) and treated with the indicated concentration of SGI-1776 for 24 h. The cell lysates were analyzed by immunoblotting with the indicated antibodies.


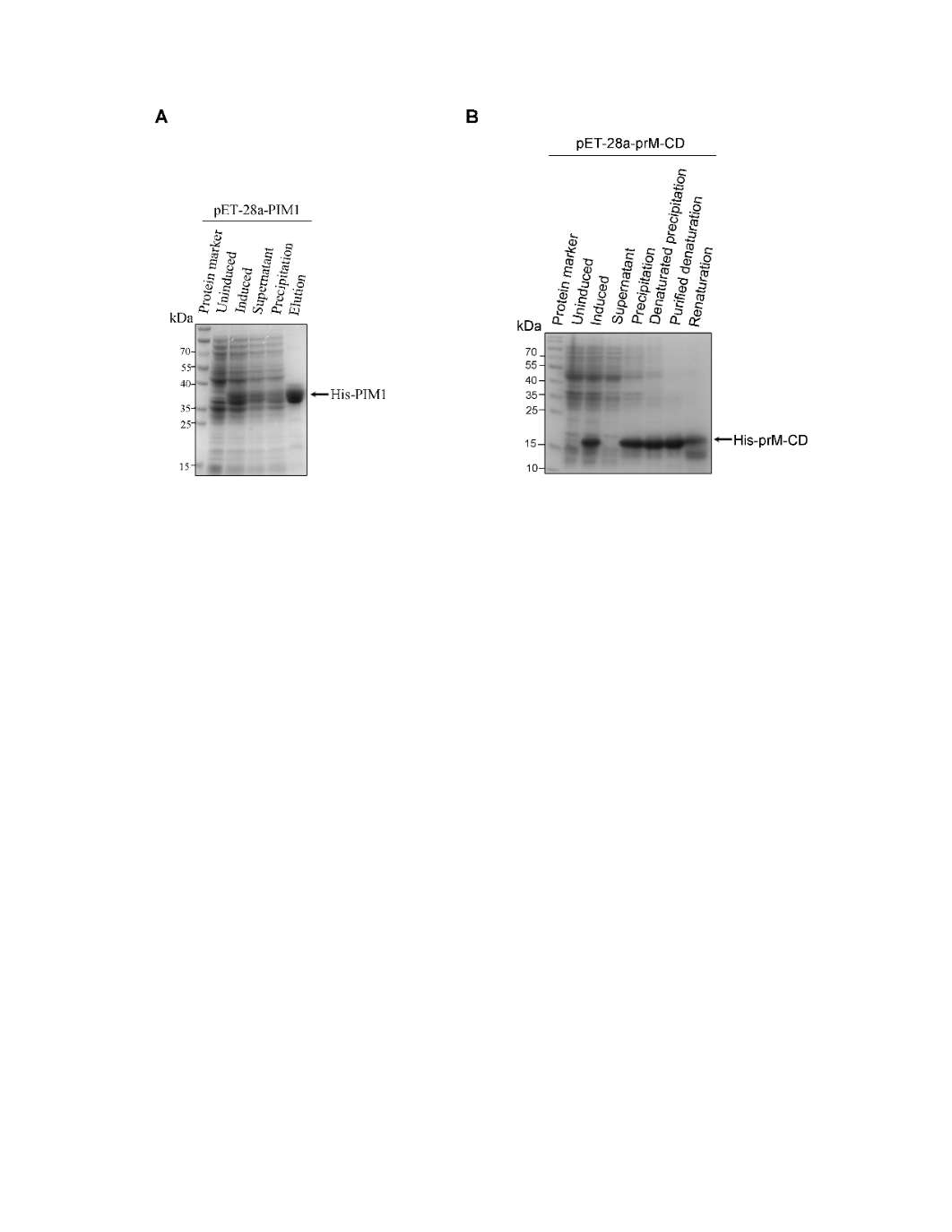


**Supplementary Figure 6.** Identification of recombinant human PIM1 and ZIKV prM via SDS‒PAGE. Figure illustrates the production and purification of recombinant human PIM1 (A) and ZIKV prM (B). The constructs expressing 6×His-PIM1 or 6×His-prM-CD were transformed into *Escherichia coli* Rosetta (DE3) competent cells and induced at the indicated temperature for the indicated time. The recombinant protein 6×His-PIM1 was purified via Ni-IDA resin (A). The inclusion body containing the recombinant protein 6×His-prM-CD was subjected to denaturation, purification with Ni-IDA resin, and renaturation to obtain the target recombinant protein (B). The indicated fractions were harvested and identified via Coomassie brilliant blue staining.


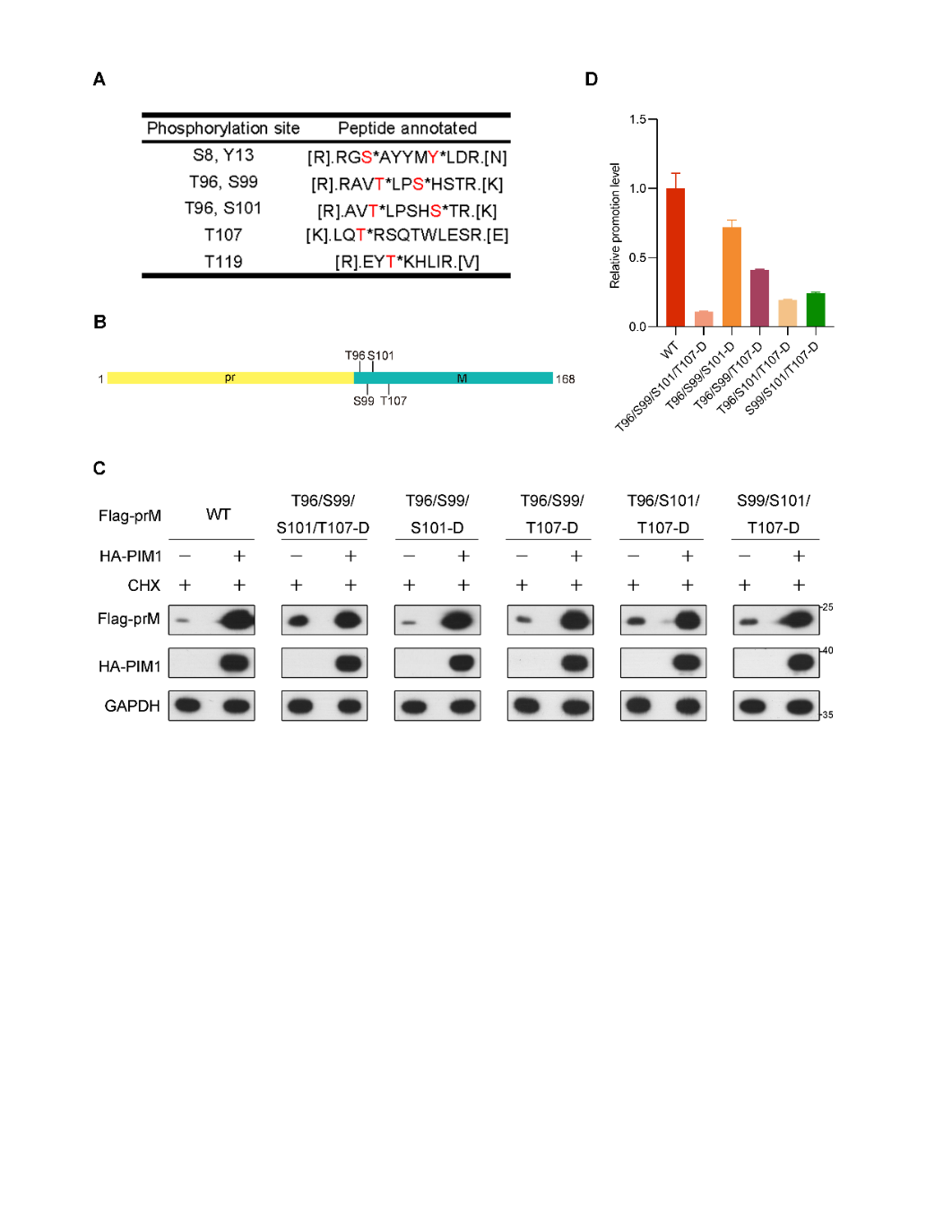


**Supplementary Figure 7.** Identification of the exact phosphorylation sites of prM catalyzed by PIM1. (**A**) Samples from the *in vitro* kinase assay were analyzed for phosphorylation sites by MS/MS. The phosphorylated sites are marked by a * in the peptide sequence. (**B**) Schematic illustration of the phosphorylation sites of prM catalyzed by PIM1. (**C**) HEK293T cells were transfected with the HA control or HA-PIM1 vector, together with the Flag-prM vector or its phosphorylation mimic mutants. At 24 h post-infection, the cells were treated with 50 μg/mL CHX for 1 h before the cellular proteins were harvested. The proteins were analyzed by immunoblotting with the indicated antibodies. (**D**) Quantification of the relative promotion levels in (C) (n = 3 independent experiments). The data are presented as the means ± SD.


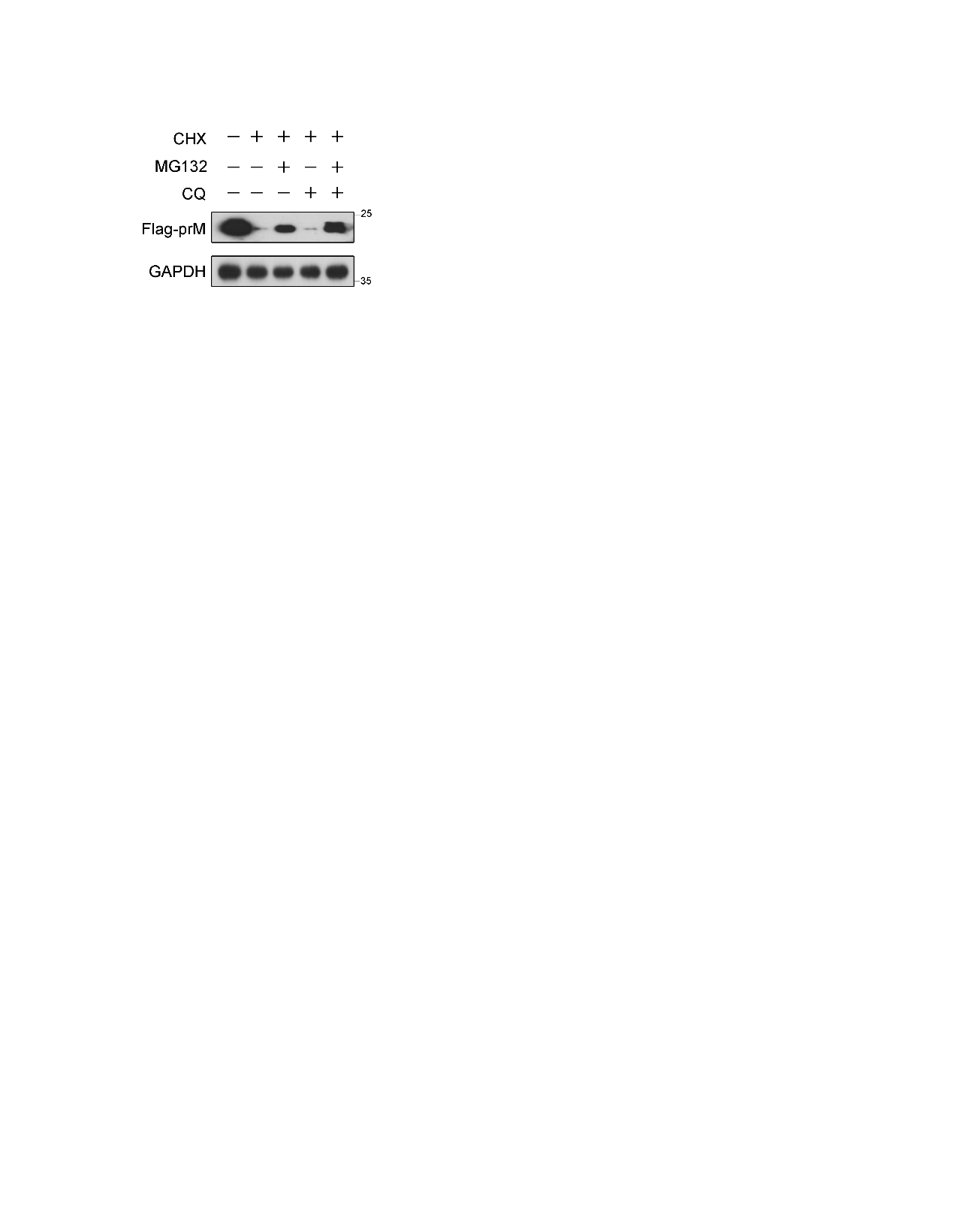


**Supplementary Figure 8.** Identification of the primary proteolytic pathway of the prM protein. A549 cells were transfected with constructs expressing Flag-prM. At 24 h after transfection, the cells were treated with 50 μg/mL CHX with or without 10 μM MG132 or 50 μM CQ for 12 h before lysis. The cell lysates were analyzed by immunoblotting with the indicated antibodies.

**
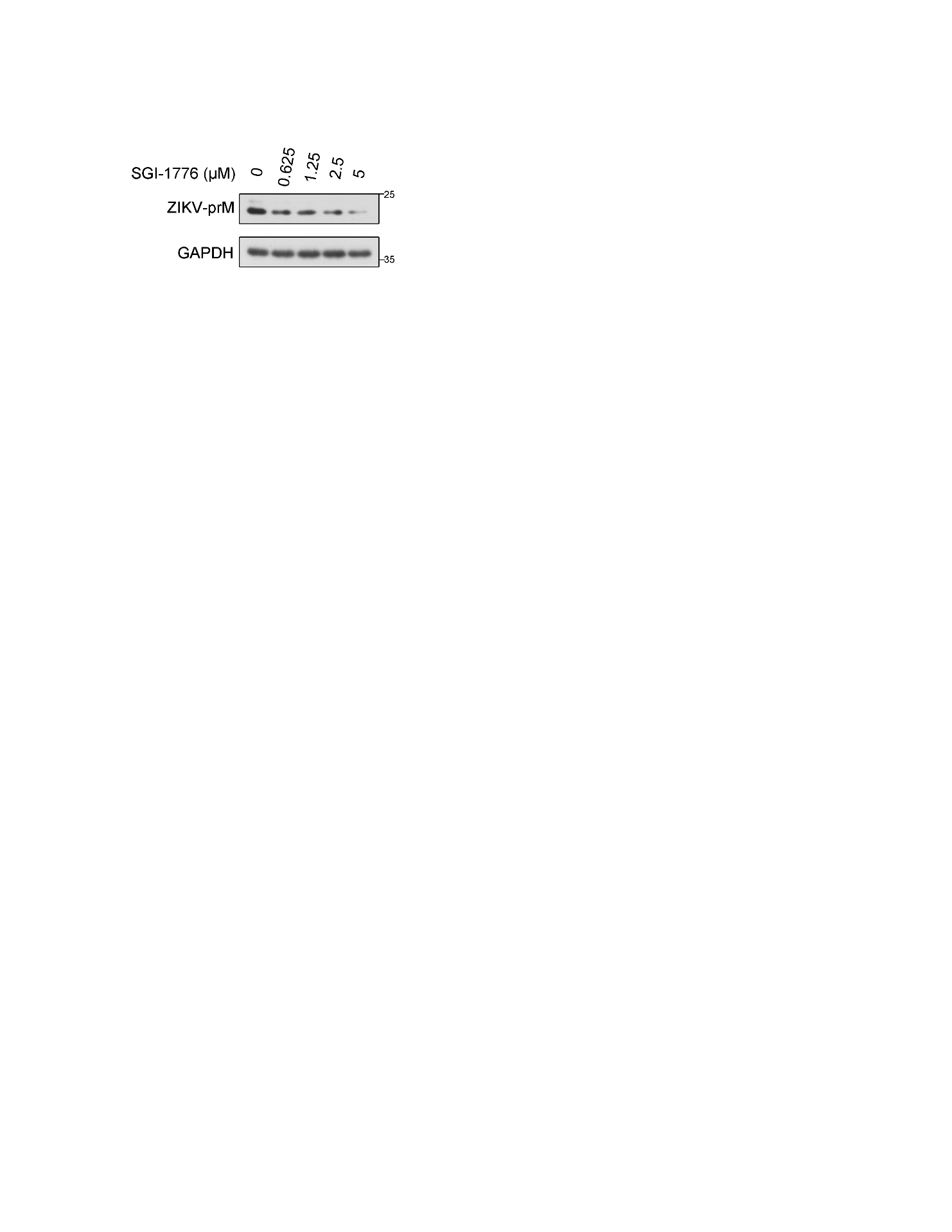
**

**Supplementary Figure 9.** Inhibition of ZIKV prM protein by SGI-1776. A549 cells were infected with ZIKV at an MOI of 0.1 and incubated with the indicated concentration of SGI-1776 for 48 h. The cell lysates were analyzed by immunoblotting with the indicated antibodies.


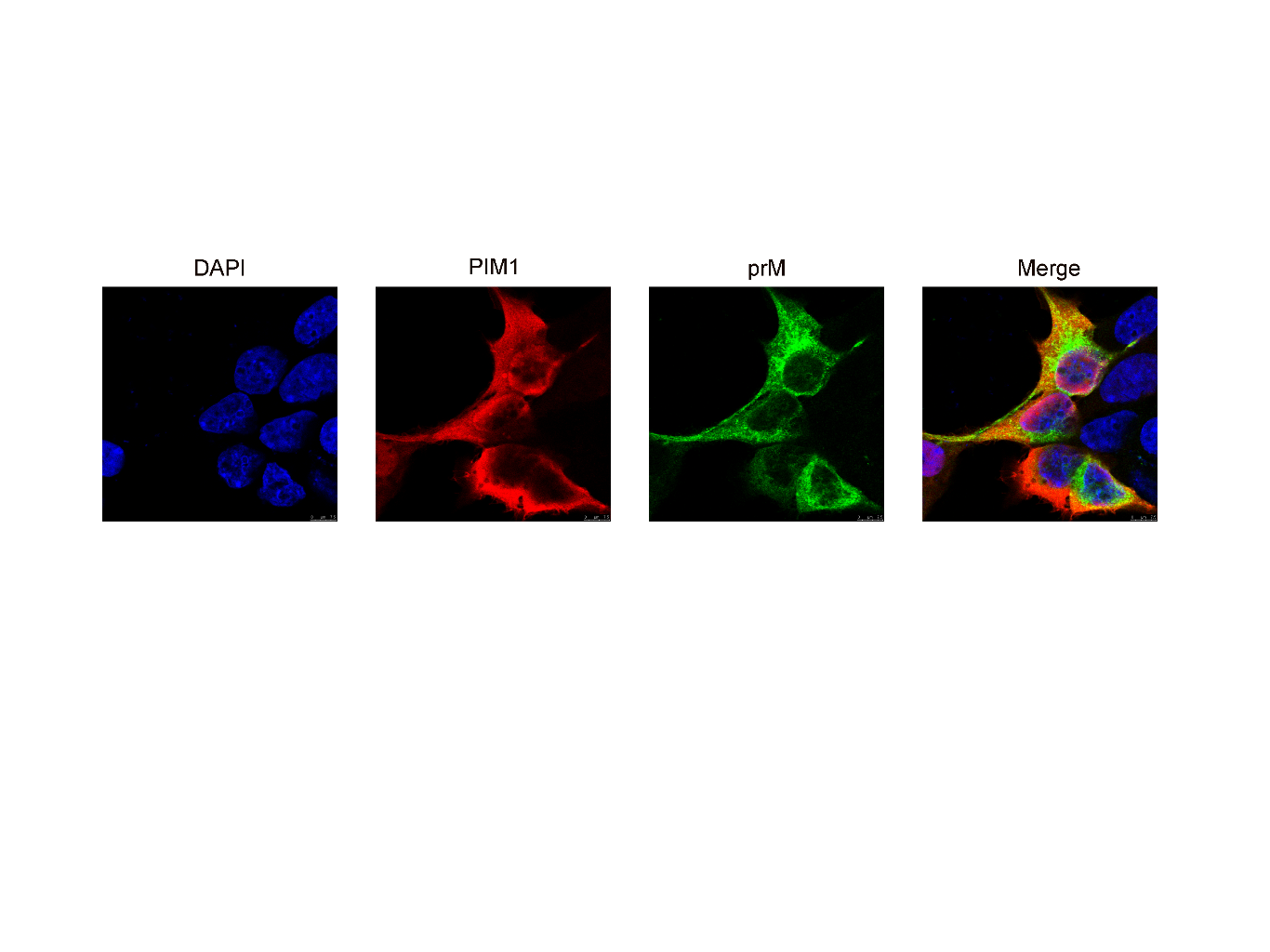


Supplementary Figure 10. Confocal imaging of PIM1 with prM. HEK293T cells transfected with constructs expressing HA-PIM1 and Flag-prM were analyzed by immunostaining with anti-HA (red) and anti-Flag (green) antibodies.
